# Niche differentiation drives microbial community assembly and succession in full-scale activated sludge bioreactors

**DOI:** 10.1038/s41522-022-00291-2

**Published:** 2022-04-11

**Authors:** Miguel de Celis, Javier Duque, Domingo Marquina, Humbert Salvadó, Susana Serrano, Lucía Arregui, Antonio Santos, Ignacio Belda

**Affiliations:** 1 Department of Genetics, Physiology and Microbiology. Unit of Microbiology. Biology Faculty, Complutense University of Madrid, 28040 Madrid, Spain; 2 Department of Evolutionary Biology, Ecology and Environmental Sciences, Faculty of Biology, Universitat de Barcelona, 08007 Barcelona, Spain

**Keywords:** Water microbiology, Microbial ecology, Water microbiology

## Abstract

Network models and community phylogenetic analyses are applied to assess the composition, structure, and ecological assembly mechanisms of microbial communities. Here we combine both approaches to investigate the temporal dynamics of network properties in individual samples of two activated sludge systems at different adaptation stages. At initial assembly stages, we observed microbial communities adapting to activated sludge, with an increase in network modularity and co-exclusion proportion, and a decrease in network clustering, here interpreted as a consequence of niche specialization. The selective pressure of deterministic factors at wastewater treatment plants produces this trend and maintains the structure of highly functional and specialized communities responding to seasonal environmental changes.

Activated sludge processes are the most applied wastewater treatment system, involving highly diverse microbial communities. The operational functioning of wastewater treatment plants (WWTPs) promotes a niche differentiation in the system, needed for the simultaneous carbon, nitrogen, and phosphorous removal, among other contaminants^1,2^. Thus, it is essential to understand the structure and assembly of these communities to ensure an optimal operation of WWTPs^3^. Most studies describing WWTP microbial communities are focused on taxonomic exploration; however, there is a need to assess which mechanisms explain the patterns arising from the interactions between taxa that shape these communities in time and space^4,5^.

The complexity of microbial communities can be analyzed through their interaction patterns, using systems approaches, like network models where nodes represent microbial taxa and edges their potential associations^6,7^. Co-occurrence networks, applied to microbial communities, include every potential association between taxa in the studied system and allow for the assessment of putative affinities to habitat preferences or ecological niches of different phylotypes^8^. Local (timepoint) networks in individual samples can be inferred from the association patterns observed at the metaweb (microbiome) level, allowing for the estimation of their individual properties to better understand community structure and dynamics, and its interaction with environmental factors^9^. In addition, the ecological processes that govern community assembly can be categorized as deterministic (abiotic and biotic factors shape patterns of community diversity and composition), and stochastic processes (changes in community structure emerge from random events, assuming all the species within a community have the same fitness)^10,11^. Recent studies suggest that both contribute to the assembly of microbial communities in WWTPs. However, there is no agreement about the importance of deterministic and stochastic processes in this system^12,13^.

In this work, we studied two timeseries, sampling over 2 years, to assess microbial community dynamics and assembly mechanisms in activated sludge bioreactors, via 16S rRNA sequencing. We compared the structure and succession patterns of the microbial communities from two municipal MBR (membrane bioreactor) WWTPs at different stages of community adaptation, in a starting up (SU-WWTP) and a fully functional plant (FF-WWTP) with similar characteristics of influent wastewater (Supplementary Fig. 1). We adapted the framework developed by Ortiz-Álvarez et al.^9^ to estimate the network properties of individual samples in a temporal scale (timepoint samples) and thus, to describe the evolution of the structure and composition of microbial communities. Then, using the approach developed by Webb^14^, we characterized the phylogenetic dispersion of co-occurring bacteria to evaluate the local relative influence of stochastic and deterministic processes in community succession.

Based on their co-occurrence patterns, the FF-WWTP metaweb was divided in two modules or communities (Fig. 1a), whereas the SU-WWTP metaweb presented three different communities (Fig. 1b). We explored the proportion of each module along the sampling period (module completeness) to study the succession patterns. We observed that the microbiome of FF-WWTP consisted of two communities alternating seasonally over the year, with different dominant communities in warmer and in colder months (correlation -Spearman- between the abundance of ASVs belonging to modules FF-1/FF-2 and temperature: R = 0.73/R = −0.62, respectively). The alternating dominance of communities at FF-WWTP seems to respond to the environmental preferences of their constituent nodes (Fig. 1c). Contrary, no seasonal pattern was observed in the module completeness of SU-WWTP metaweb, but a consistent evolution of community composition where module SU-3 decreased in favor of the other two modules (Fig. 1d). It should be noted that an exogenous community (activated sludge), coming from another similar MBR-WWTP was used as seeding inoculum to start the operation of SU-WWTP (unfortunately, there is no further information about the composition of the exogenous community). Thus, we argue that the pattern showed by SU-WWTP responds to the early adaptation stages of community assembly, whereas FF-WWTP showed the population dynamics of an established community. Similarly, we studied the abundance of nodes correlating with pollutant removal rates (Supplementary Table 1). Just a few nodes of the FF-WWTP metaweb correlated with nutrients removal rates (Supplementary Fig. 2b), and the subtle temporal patterns observed in nodes correlating with total nitrogen (TN) and biological oxygen demand (BOD) removal rates (Fig. 1e) can be indirectly explained by the increased influent concentrations of these pollutants during the warmer months (Supplementary Fig. 1), when phylotypes of module FF-1 appeared as dominating. However, a clear pattern is observed in SU-WWTP, where there is a net increase in TN and total phosphorous (TP) removal rates from the beginning to the end of the sampling period (Supplementary Fig. 1), accompanied by the increase in abundance of nodes positively correlated with TN and TP removal rates (Fig. 1f). This pattern corresponds with the switch in the dominance of the SU-3 module in favor of two arising communities (modules SU-1 and SU-2) positively correlated with TN and TP removal rates (Supplementary Fig. 2d). Interestingly, the taxonomic affiliation of these correlating phylotypes reveals several bacterial genera related to nitrogen and phosphorous removal in the disappearing community (e.g., *Accumulibacter*, *Haliangium*, *Nitrospira*, *Nitrosomonas*, *Pirellula*, among others) (Supplementary Table 1). We hypothesize that these strains, possibly coming from the seeding community used as inoculum in SU-WWTP, may be not adapted to this new environment, and thus are disappearing in favor of more adapted strains, which can perform more efficiently the depuration processes in this WWTP (e.g., *Nitrosomonas*, *Pirellula*, among others in the module SU-1). Besides, we found a conserved taxonomical pattern at the Phylum level between modules in both WWTPs (Student’s *t*-test, *p* < 0.05), showing that the abundance of Firmicutes is lower in modules with more nodes positively correlated with nutrient removal, while Planctomycetes has higher abundance in these modules (Supplementary Fig. 2).

**Fig. 1:**
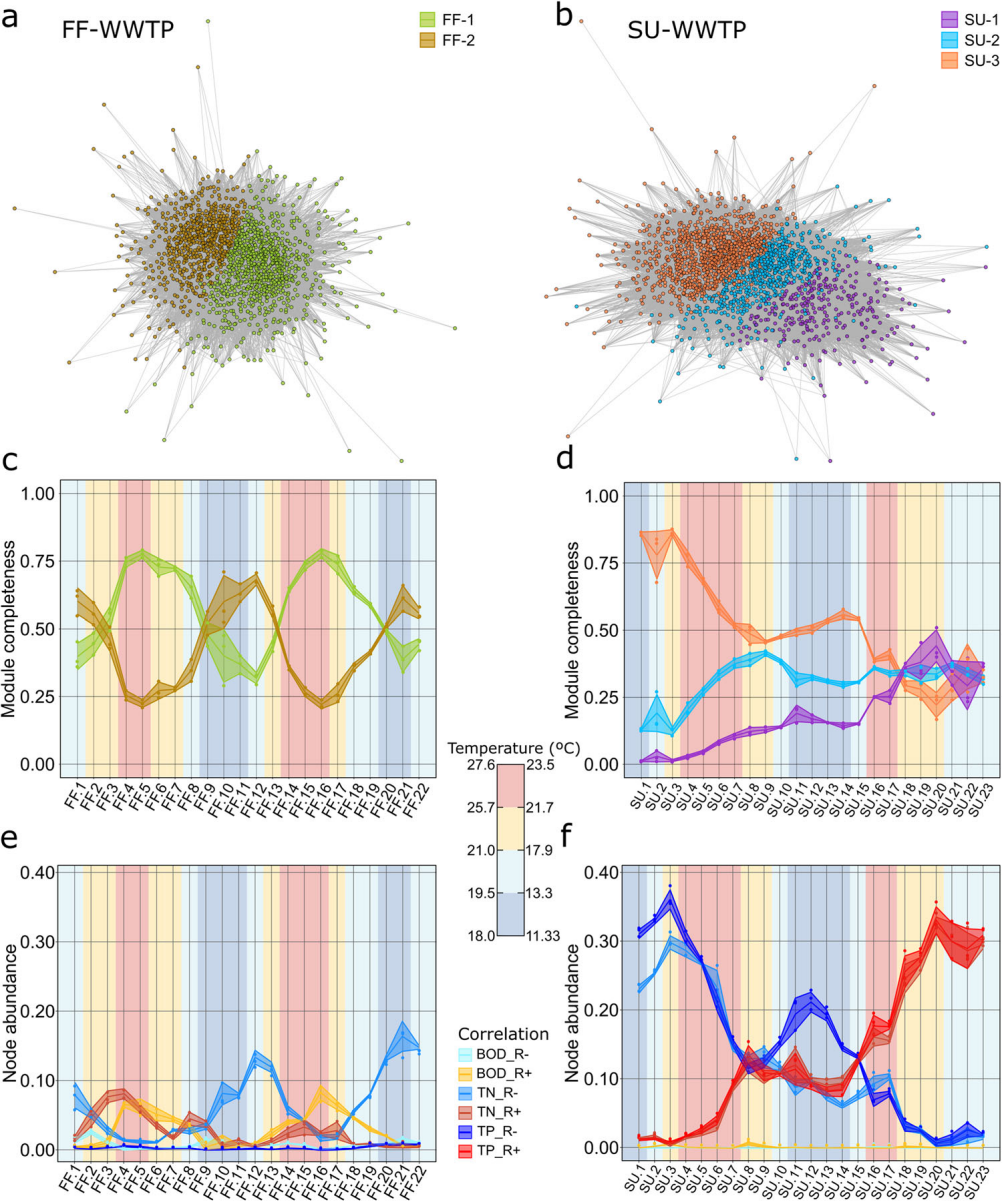
Ecological clusters based on co-occurrence patterns. The left panels represent data from the fully functional plant (FF-WWTP), and the right panels represent the starting up plant (SU-WWTP). Network nodes represent community members (ASV) and edges significant relationships between nodes (probability of co-occurrence higher than 0.95). **a**, **b** Nodes are colored based on their module membership, being FF-WWTP divided in two different modules (FF-1, green and FF-2, brown) and SU-WWTP in three (SU-1, purple; SU-2, blue; and SU-3, orange). **c,** **d** Module completeness, the proportion of nodes belonging to a given module present in each individual sample. **e**, **f** Abundance of nodes correlated to any given pollutant removal rate. The central lines correspond to average values (*n* = 3) of each sample, and the shaded areas correspond to the standard deviation. The background of the plots is colored based on the temperature measured when collecting samples. Temperature data were divided in quartiles (between 18.0 and 27.6 °C for FF-WWTP and, between 11.33 and 23.50 °C for SU-WWTP) for a simpler representation. Temporal sampling frame includes a 2-year monthly sampling from April 2017 to March 2019 (August not sampled) for FF-WWTP and from March 2017 to February 2019 (June 2019 not sampled) for SU-WWTP.

Then, we inferred the timepoint networks of each individual sample from the co-occurrence/co-exclusion metawebs, providing monthly snapshots of the microbiome network. Despite the marked seasonal effect found in community composition of FF-WWTP (Fig. 1b and Supplementary Fig. 3a), there is no clear temporal pattern on its timepoint network properties (Fig. 2a, Supplementary Fig. 4a, c), in agreement with the findings of Sun et al.^15^ studying networks’ topology in fully functional WWTPs. On the contrary, in the SU-WWTP a steady increase in modularity and co-exclusion proportion was accompanied with a decrease in clustering coefficient (Fig. 2b; see Supplementary Fig. 4b, d for data normalized to null values), possibly reflecting the niche specialization the community is going towards^16^. In both WWTPs, clustering coefficient showed a negative correlation with modularity and co-exclusion proportion (Supplementary Table 2). These relationships may suggest a conserved response of low specialized and interconnected communities to environmental gradients in a succession towards a stronger niche specialization^9,17^. Thus, SU-WWTP microbiome assembly shows a transition between low modular and aggregated (clustered) communities, resembling small-world networks^18^, to highly modular low-clustered communities with differentiated niches.

**Fig. 2:**
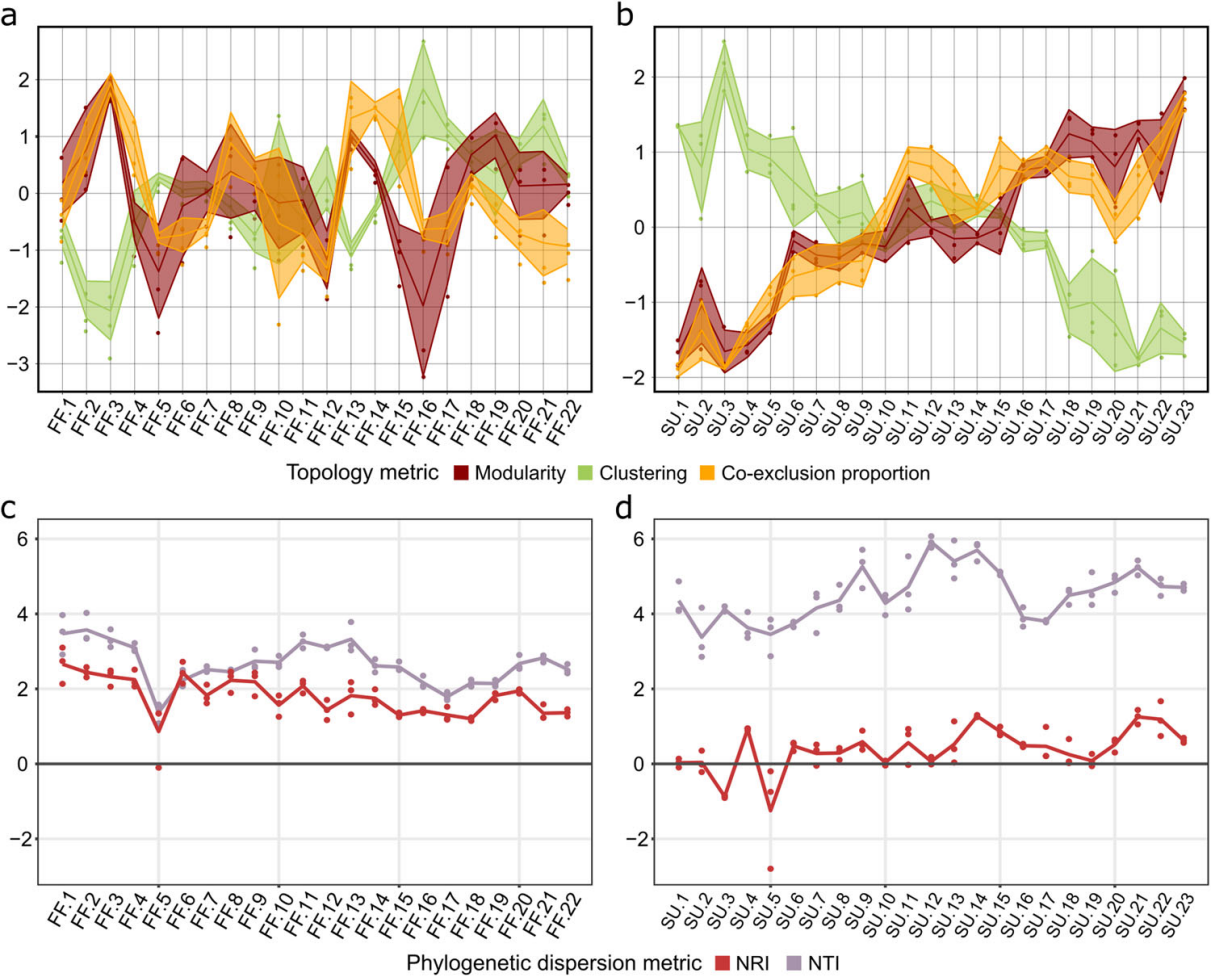
Evolution of bacterial community structure and ecological processes shaping community assembly and succession. Left panels correspond to FF-WWTP and right panels to SU-WWTP. **a,** **b** Temporal patterns of modularity and clustering coefficients, and nodes co-exclusion proportion are represented in red, green, and orange, respectively. The central line corresponds to average values (*n* = 3) of each sample, and the shaded areas correspond to the standard deviation. **c,** **d** Community assembly assessed via net relatedness index (NRI, dark red) and nearest taxon index (NTI, purple). Dots represent sample data (*n* = 3) and lines represent the average value per timepoint.

Finally, we assessed the phylogenetic dispersion of the communities calculating the net relatedness index (NRI), which examines the clustering/dispersion within an assemblage, and nearest taxon index (NTI), which examines the dispersion of closely related phylotypes^14^. We found significant phylogenetic signals across relatively short phylogenetic distances (Supplementary Fig. 5), justifying the use of phylogenetic null models in this study^19^. These measures are relative to a shared species pool used to build the phylogeny; thus, we can only compare them within each WWTP^14^. NTI values (FF-WWTP: 2.666 ± 0.583; SU-WWTP: 4.516 ± 0.759) were higher than NRI values (FF-WWTP: 1.798 ± 0.536; SU-WWTP: 0.378 ± 0.665) in both WWTPs (Fig. 2c, d and Supplementary Table 3), with a greater difference in SU-WWTP, revealing that niche conservatism is more relevant at terminal levels in the phylogeny^20^, as reflected by the phylogenetic signal. Highly positive NTI values reveal significant phylogenetic clustering (underdispersion) and, based on the assumption that phylogenetically similar groups share similar niches, this indicates deterministic habitat filtering^19,21,22^. Besides, we observed increasing trends in NRI and NTI values in SU-WWTP, suggesting that this community is going towards a higher phylogenetic clustering, indicating the increasing importance of deterministic processes over time at early stages of community assembly. The phylogenetic clustering revealed by the NTI can be interpreted as environmental filtering^23^, while the increasing NRI trend in SU-WWTP suggests greater changes in community composition, as they can be detected across the entire phylogeny (also revealed by β-diversity, Supplementary Fig. 3b). The clustering measured by NTI, and the random distribution measured by NRI, indicates that close phylotypes from few disparate lineages co-occur in the early stages of SU-WWTP succession. However, in the FF-WWTP we observed a slighter decreasing pattern in NRI, which could be revealing an effect of drift in the succession of this community over the sampling period (Supplementary Fig. 3a), while maintaining the functional structure in response to the habitat filtering and the seasonal environmental dynamics within the bioreactor^24^. Furthermore, we found a decreasing trend in α-diversity in the SU-WWTP (Supplementary Fig. 6 and Supplementary Table 3) accounting for relative abundance and richness (^2^D, ^1^D, and ^0^D), as well as at the phylogenetic and taxonomic level (^0^pD and ^0^D). We also found negative correlations (Spearman) between α-diversity measures (^1^D, ^0^D, and ^0^pD) and NTI, but not NRI (Supplementary Fig. 6 and Supplementary Table 3). This trend reveals the environmental filtering processes dominating SU-WWTP community succession^23^, which leads to lower α-diversity values, similar to those found in FF-WWTP.

In this work, we integrate information from microbiome and timepoint networks analyses, and phylogenetic information to assess community associations, structure, and assembly processes. The ordination plot of beta diversity of FF-WWTP showed a seasonal succession (Supplementary Fig. 3a) in agreement with the two alternating communities observed with module completeness (Fig. 1). By the time of sampling, FF-WWTP microbiome was already adapted to the wastewater environment, and the alternating communities responded to the habitat preferences of their phylotypes, mainly defined by temperature variations^24^. However, the SU-WWTP microbiome was on its early adaptation to the WWTP environment, and its community dynamics showed a constant non-cyclical succession, subtly affected by seasonal environmental changes (Supplementary Fig. 3b). The dynamics described by network properties, module completeness and phylogenetic dispersion, suggest that the assembly and succession patterns of microbial communities at both WWTPs are governed by deterministic processes that select for phylogenetically similar communities^22^. We also observed the increasing importance of environmental filtering processes in the early stages of SU-WWTP community assembly. This could be explained by the increasing importance of biotic filtering during succession^25^, leading to a phylogenetic dispersion similar to FF-WWTP. The calculation of timepoint networks allowed us to detect the effect of deterministic processes, inferred with metrics based on null models of α-diversity, in the community composition (module completeness) and structure (network topologies). In this context, we hypothesize that the inverse relationship between the steady increase in modularity and the decrease in clustering coefficient may reflect the niche differentiation affecting the community assembly. In a system where population dynamics responds to niche-driven selection, competition may play an important role in microbial survival over time, favoring those strains with increased fitness in the specific wastewater and environmental conditions of the WWTP^26^. Thus, we argue that highly specialized and competitive communities emerge on activated sludge bioreactors, in a succession governed by deterministic processes.

## Methods

### Sample collection and basic wastewater characterization

Activated sludge samples were collected over a period of 2 years from two full-scale municipal WWTPs from Spain treating residential wastewater, equipped with a membrane bioreactor system (MBR), one fully functional (FF-WWTP, started operating in 1986) and one starting up plant (SU-WWTP, started operating at the time of starting the sampling). The samples were taken monthly and consisted of mixed liquor collected from the three functional stages in which the bioreactor was divided, acting as triplicates due to the wastewater recirculation in the system. FF-WWTP samples were taken from April ’17 to March ’19 (August months were not sampled, thus the time between samples FF-4/FF5 and FF-15/FF-16 is 2 months instead of one) accounting for a total of 22 sampling times (*n* = 3 per sampling time). SU-WWTP samples were taken from March ’17 to February ’19 (June ’18 was not sampled, thus the time between samples FF-15/FF-16 is 2 months instead of one), accounting for a total of 23 sampling times (*n* = 3 per sampling time). Physical-chemical parameters were measured in the influent and the effluent of the WWTP, according to standard methods: Biochemical Oxygen Demand, BOD (UNE-EN-1899); Total Nitrogen, TN (ISO-11905) and Total Phosphorous, TP (ISO-6878). The removal rate (_R) was calculated accordingly. Detailed information concerning plant physical-chemical and operational parameters is summarized in Supplementary Table 3.

### DNA extraction, sequencing, and amplicon reads processing

DNA extraction, sequencing and reads processing was performed as previously described^24^. Briefly, DNA was extracted using DNA Power Soil extraction kits, libraries were prepared following the two-step PCR Illumina® protocol and sequenced on Illumina MiSeq instrument (Illumina®, San Diego, CA, USA) using 2 × 300 paired-end reads. The 16S-V4 rRNA gene was amplified with the primer set: 515 F -GTGYCAGCMGCCGCGGTAA- and 806 R -GGACTACNVGGGTWTCTAAT-. Dada2 algorithm^27^ implemented in R pipeline was used to perform sequence analysis, such as denoise, filter, align pairs and filter out chimeras. A total of 3,575,184 (54,169 from 66 samples in total) and 3,924,039 (56,870 from 69 samples) good quality reads and 6245 and 9837 amplicon sequences variants (ASVs) were obtained for FF-WWTP and SU-WWTP, respectively. The taxonomic assignment was performed using the naïve Bayesian classifier implemented in Dada2 using as reference database Silva (release 132), with a bootstrap cutoff of 80%, and using the SINTAX algorithm^28^ based on the MiDAS database v.4.8.1^29^. Besides, we used Hill diversity indices to quantify taxonomic and phylogenetic α-diversity^30^ using the hillR R package^31^. Community structure was evaluated through a non-metric multidimensional (NMDS) ordination of Bray-Curtis dissimilarity matrices (Supplementary Data 1), constructed from abundance data using the vegan R package^32^.

### Network models and topological properties calculation

Samples were filtered by occurrence keeping ASVs present in more than one, but not in every timepoint, resulting in 2322 and 3348 ASVs from FF-WWTP and SU-WWTP, respectively. We calculated every potential co-occurrences and co-exclusions between nodes applying a probabilistic model^33^, obtaining a list of significant co-occurring and co-excluding pairs (Supplementary Data 1). Co-occurrence/co-exclusion regional metawebs consisted of a total of 1493/1789 and 1879/2290 nodes, for the FF-WWTP and SU-WWTP, respectively. We used the function cluster_waltrap from the igraph R package^34^ to determine the module of the co-occurrence regional metawebs in which each node belongs. Then, we inferred the individual networks of each time-step sample from the co-occurrence/co-exclusion regional metawebs. We extracted the nodes corresponding to ASV present in each sample and the links between them and constructed undirected time-step networks with igraph^34^. From each time-step co-exclusion networks we calculated the co-exclusion proportion, while modularity and clustering coefficient were calculated from each time-step co-occurrence networks. We applied a stochastic block model to generate networks with the same properties as the observed in both WWTPs. Then, we simulated the timepoint networks picking the same number of nodes from each module that in the observed networks (1000 iterations). Finally, we calculated null distributions for each network property. Besides, we calculated the Spearman’s rank correlations between the abundance of co-occurring nodes and the nutrient removal rates (total nitrogen, total phosphorous, and biological oxygen demand removal rates, Supplementary Data 2), correcting *p*-values with false discovery rate (FDR, 5%).

### Microbial community phylogenetic dispersion against a null expectation

We applied a null model on phylogenetic α-diversity framework to assess the influence of stochastic or deterministic assembly processes^14^. These phylogenetic null-model metrics are relevant when there is phylogenetic signal, when closely related ASVs have similar niches^19^. First, we calculated the abundance-weighted mean of the selected environmental parameters (Influent, effluent, and operational parameters and environmental characteristics Supplementary Data 2) for each ASV (i.e., ASV environmental optima). Then, we calculated the pairwise ASV phylogenetic distance. To do so, we aligned the sequences with the msa R package^35^ and used the phangorn R package^36^ to construct a phylogenetic tree and fit a GTR + G + I maximum likelihood tree. Then, we calculated the pairwise distances between each pairs of tips from the phylogenetic tree using its branch lengths, using the cophenetic function of the ape R package^37^. Finally, the phylogenetic signal was tested using Mantel correlograms between ASV environmental optima and ASV phylogenetic distances. We found significant phylogenetic signal across short phylogenetic distance (Supplementary Fig. 5), indicating that the environmental preferences of ASVs were phylogenetically conserved across relatively short phylogenetic distances^33^. First, we calculated the net relatedness index (NRI), which is −1 times the standardized effect size (SES) of the mean pairwise diversity (MPD) and measures the dispersion across the entire phylogenetic tree. Then, we calculated the nearest taxon index (NTI), which is negative of the SES of the mean nearest taxon distance (MNTD) and measures the dispersion of closely related ASVs^38^. SES of MPD and MNTD were calculated with the ses.mpd and ses.mntd functions (null.model = “taxa.labels”, abundance.weighted = TRUE) of the picante R package^39^. The closer to zero NRI or NTI are, the closer the phylogenetic structure of the community is to the null expectation (higher stochasticity). When values are below zero, these metrics describe the reflect phylogenetic overdispersion, with high influence of biological interaction, and when values are above zero, reflect phylogenetic clustering caused by deterministic habitat filtering^20^.

### Reporting summary

Further information on research design is available in the Nature Research Reporting Summary linked to this article.

## Supplementary information

SUPPLEMENTAL MATERIAL

Supplementary data 1

Supplementary data 2

Reporting Summary Checklist

## Data Availability

Raw files are available in the National Center for Biotechnology (NCBI) repository under the project codes PRJNA588045 for FF-WWTP sequences and PRJNA719992 for SU-WWTP sequences.
