# Supplementary material for: Niche differentiation drives microbial community assembly and succession in full-scale activated sludge bioreactors
Source: NPJ Biofilms Microbiomes. 2022 Apr 11;8:23. doi: 10.1038/s41522-022-00291-2 (PMC9001656; doi:10.1038/s41522-022-00291-2)
Supplement: Supplementary file 1 — SUPPLEMENTAL MATERIAL [file 41522_2022_291_MOESM1_ESM.pdf]

SUPPLEMENTARY TABLE 1. Nodes significantly correlated (FDR < 0.05) to a pollutant degradation rate (BOD: biological oxygen demand; TN: total nitrogen; TP: total phosphorous). On each pollutant column is indicated the significant Spearman's rank correlation between the node abundance and the degradation rate.

WWTP	Community	Main Pollutant	BOD_R	TN_R	TP_R	Genus (MiDaS)
FF-WWTP	2	BOD_R-	-0.5168			Unidentified
FF-WWTP	2	BOD_R-	-0.4415			Blastopirellula
FF-WWTP	2	BOD_R-	-0.4110			Unidentified
FF-WWTP	1	BOD_R+	0.4145			Unidentified
FF-WWTP	1	BOD_R+	0.4147			Unidentified
FF-WWTP	1	BOD_R+	0.4184			Byssovorax
FF-WWTP	1	BOD_R+	0.4211			Pir4_lineage
FF-WWTP	1	BOD_R+	0.4233			Unidentified
FF-WWTP	1	BOD_R+	0.4243			Unidentified
FF-WWTP	1	BOD_R+	0.4273			Unidentified
FF-WWTP	1	BOD_R+	0.4283			Unidentified
FF-WWTP	1	BOD_R+	0.4338			Unidentified
FF-WWTP	1	BOD_R+	0.4433			Halomonas
FF-WWTP	1	BOD_R+	0.4450			Unidentified
FF-WWTP	1	BOD_R+	0.4455			Unidentified
FF-WWTP	1	BOD_R+				Pirellula
FF-WWTP	1	BOD_R+	0.4480			Unidentified
FF-WWTP	1	BOD_R+	0.4514			Unidentified
FF-WWTP	1	BOD_R+	0.4665			Unidentified
FF-WWTP	1	BOD_R+	0.4783			JGI_0001001-H03
FF-WWTP	1	BOD_R+	0.4847			Unidentified
FF-WWTP	1	BOD_R+	0.5058			Acinetobacter
FF-WWTP	1	BOD_R+	0.5204			Unidentified
FF-WWTP	1	BOD_R+	0.5303			Unidentified
FF-WWTP	1	BOD_R+	0.5682			Alkanindiges
FF-WWTP	2	TN_R-		-0.5645		Unidentified
FF-WWTP	2	TN_R-		-0.5494		Unidentified
FF-WWTP	2	TN_R-		-0.5373		Unidentified
FF-WWTP	2	TN_R-		-0.4911		Flavobacterium
FF-WWTP	2	TN_R-		-0.4903		Pedomicrobium
FF-WWTP	2	TN_R-		-0.4900		Unidentified
FF-WWTP	1	TN_R-		-0.4846		Unidentified
FF-WWTP	2	TN_R-		-0.4833		Mesorhizobium
FF-WWTP	2	TN_R-		-0.4823		Unidentified
FF-WWTP	2	TN_R-		-0.4813		Unidentified
FF-WWTP	2	TN_R-		-0.4730		Runella
FF-WWTP	2	TN_R-		-0.4658		Unidentified
FF-WWTP	2	TN_R-		-0.4652		Unidentified
FF-WWTP	2	TN_R-		-0.4580		Pirellula
FF-WWTP	2	TN_R-		-0.4506		DMER64

FF-WWTP	2	TN_R-	-0.4473	Unidentified
FF-WWTP	2	TN_R-	-0.4468	SH3-11
FF-WWTP	2	TN_R-	-0.4377	Ca_Microthrix
FF-WWTP	2	TN_R-	-0.4327	Unidentified
FF-WWTP	2	TN_R-	-0.4317	Christensenellaceae_R-7_group
FF-WWTP	2	TN_R-	-0.4235	Unidentified
FF-WWTP	2	TN_R-	-0.4189	BD1-7_clade
FF-WWTP	2	TN_R-	-0.4170	Unidentified
FF-WWTP	2	TN_R-	-0.4157	Unidentified
FF-WWTP	2	TN_R-	-0.4148	Ca_Sarcinithrix
FF-WWTP	2	TN_R-	-0.4133	Unidentified
FF-WWTP	2	TN_R-	-0.4085	Unidentified
FF-WWTP	2	TN_R-	-0.4013	Unidentified
FF-WWTP	2	TN_R-	-0.3958	Unidentified
FF-WWTP	2	TN_R-	-0.3955	Unidentified
FF-WWTP	2	TN_R-	-0.3889	Sphingorhabdus
FF-WWTP	2	TN_R-	-0.3884	Unidentified
FF-WWTP	2	TN_R-	-0.3884	Paracoccus
FF-WWTP	2	TN_R-	-0.3859	SH-PL14
FF-WWTP	2	TN_R-	-0.3845	Cytophaga
FF-WWTP	2	TN_R-	-0.3844	Unidentified
FF-WWTP	2	TN_R-	-0.3799	Unidentified
FF-WWTP	2	TN_R-	-0.3768	Unidentified
FF-WWTP	2	TN_R-	-0.3754	Unidentified
FF-WWTP	2	TN_R-	-0.3734	CL500-29_marine_group
FF-WWTP	2	TN_R-	-0.3730	Unidentified
FF-WWTP	2	TN_R-	-0.3717	Unidentified
FF-WWTP	2	TN_R-	-0.3701	Unidentified
FF-WWTP	2	TN_R-	-0.3667	Unidentified
FF-WWTP	2	TN_R-	-0.3666	Unidentified
FF-WWTP	1	TN_R-	-0.3664	Unidentified
FF-WWTP	2	TN_R-	-0.3660	SM1A02
FF-WWTP	1	TN_R-	-0.3648	Terrimonas
FF-WWTP	1	TN_R+	0.3693	Oikopleura
FF-WWTP	1	TN_R+	0.3708	Unidentified
FF-WWTP	1	TN_R+	0.3788	Ellin6067
FF-WWTP	1	TN_R+	0.3790	Unidentified
FF-WWTP	1	TN_R+	0.3793	Chitinivorax
FF-WWTP	1	TN_R+	0.3827	Unidentified
FF-WWTP	1	TN_R+	0.3872	Unidentified
FF-WWTP	1	TN_R+	0.3877	Unidentified
FF-WWTP	1	TN_R+	0.3891	Unidentified
FF-WWTP	2	TN_R+	0.3920	Unidentified
FF-WWTP	1	TN_R+	0.4005	Unidentified
FF-WWTP	1	TN_R+	0.4022	Unidentified

FF-WWTP	2	TN_R+		0.4064		Unidentified
FF-WWTP	1	TN_R+		0.4074		Unidentified
FF-WWTP	1	TN_R+		0.4089		Unidentified
FF-WWTP	1	TN_R+		0.4129		Unidentified
FF-WWTP	2	TN_R+		0.4190		Oikopleura
FF-WWTP	1	TN_R+		0.4264		Unidentified
FF-WWTP	1	TN_R+		0.4306		Ellin6067
FF-WWTP	1	TN_R+		0.4316		Subgroup_10
FF-WWTP	1	TN_R+		0.4333		Unidentified
FF-WWTP	1	TN_R+		0.4441		Unidentified
FF-WWTP	1	TN_R+		0.4479		Unidentified
FF-WWTP	1	TN_R+		0.4482		Unidentified
FF-WWTP	1	TN_R+		0.4569		Unidentified
FF-WWTP	1	TN_R+		0.4804		Unidentified
FF-WWTP	1	TN_R+		0.4888		Phaeodactylibacter
FF-WWTP	1	TN_R+		0.4962		Unidentified
FF-WWTP	1	TN_R+		0.5318		Dechloromonas
FF-WWTP	1	TN_R+		0.5357		Fluviicoccus
FF-WWTP	1	TN_R+		0.5759		Agitococcus_lubricus_group
FF-WWTP	2	TP_R-			-0.5122	Unidentified
FF-WWTP	1	TP_R-			-0.5009	Rhodopirellula
SU-WWTP	3	TP_R-		-0.6014	-0.7529	Unidentified
SU-WWTP	3	TP_R-		-0.3531	-0.6588	Fimbriiglobus
SU-WWTP	3	TP_R-		-0.6252	-0.6553	Unidentified
SU-WWTP	3	TP_R-		-0.3792	-0.6373	Unidentified
SU-WWTP	3	TP_R-		-0.4747	-0.6252	Unidentified
SU-WWTP	3	TP_R-		-0.6112	-0.6252	Unidentified
SU-WWTP	2	BOD_R-	-0.6110			Unidentified
SU-WWTP	3	TP_R-		-0.5799	-0.6063	Unidentified
SU-WWTP	3	TP_R-		-0.3985	-0.6004	Ferruginibacter
SU-WWTP	3	TP_R-		-0.5720	-0.5975	Unidentified
SU-WWTP	3	TP_R-		-0.5402	-0.5956	Unidentified
SU-WWTP	3	TN_R-		-0.5950	-0.4608	Unidentified
SU-WWTP	3	TN_R-		-0.5943	-0.4950	Unidentified
SU-WWTP	3	TP_R-		-0.3385	-0.5865	Nannocystis
SU-WWTP	3	TP_R-			-0.5834	Terrimonas
SU-WWTP	3	TP_R-			-0.5818	Unidentified
SU-WWTP	3	TP_R-		-0.3954	-0.5801	Gordonia
SU-WWTP	3	TP_R-		-0.3280	-0.5792	Unidentified
SU-WWTP	3	TP_R-		-0.5413	-0.5769	Lewinella
SU-WWTP	3	TP_R-		-0.5475	-0.5759	Unidentified
SU-WWTP	3	TN_R-		-0.5755	-0.5499	Ca_Berkiella
SU-WWTP	3	TP_R-		-0.4760	-0.5740	Unidentified
SU-WWTP	3	TP_R-		-0.4425	-0.5733	Fimbriiglobus
SU-WWTP	3	TP_R-		-0.3869	-0.5723	Unidentified
SU-WWTP	3	TP_R-			-0.5691	Terrimonas

SU-WWTP	3	TP_R-	-0.4800	-0.5667	Unidentified
SU-WWTP	3	TP_R-	-0.5532	-0.5661	Flavobacterium
SU-WWTP	3	TP_R-	-0.4703	-0.5621	Kouleothrix
SU-WWTP	3	TN_R-	-0.5554	-0.3874	Unidentified
SU-WWTP	3	TN_R-	-0.5535	-0.4825	Unidentified
SU-WWTP	3	TN_R-	-0.5528	-0.5290	Pirellula
SU-WWTP	3	TP_R-	-0.4714	-0.5483	Unidentified
SU-WWTP	3	TP_R-	-0.5291	-0.5464	Unidentified
SU-WWTP	3	TP_R-	-0.4612	-0.5457	Ellin6067
SU-WWTP	3	TN_R-	-0.5443	-0.5441	Unidentified
SU-WWTP	3	TP_R-	-0.4109	-0.5431	Unidentified
SU-WWTP	3	TP_R-	-0.5321	-0.5407	Unidentified
SU-WWTP	3	TP_R-	-0.3925	-0.5360	Unidentified
SU-WWTP	3	TN_R-	-0.5352	-0.4999	Unidentified
SU-WWTP	3	TN_R-	-0.5350	-0.3782	Unidentified
SU-WWTP	3	TP_R-	-0.4136	-0.5339	Unidentified
SU-WWTP	3	TP_R-	-0.5132	-0.5335	Unidentified
SU-WWTP	3	TN_R-	-0.5332	-0.5060	Unidentified
SU-WWTP	3	TP_R-	-0.5071	-0.5324	Unidentified
SU-WWTP	3	TP_R-	-0.5305	-0.5320	Unidentified
SU-WWTP	3	TP_R-	-0.4880	-0.5297	Rhodobacter
SU-WWTP	3	TN_R-	-0.5279	-0.4863	Unidentified
SU-WWTP	3	TP_R-	-0.4029	-0.5263	Unidentified
SU-WWTP	3	TP_R-	-0.4485	-0.5261	Unidentified
SU-WWTP	3	TP_R-	-0.3377	-0.5251	Unidentified
SU-WWTP	3	TP_R-	-0.4586	-0.5208	Unidentified
SU-WWTP	3	TP_R-	-0.4701	-0.5198	Ellin6067
SU-WWTP	3	TP_R-	-0.4078	-0.5196	Nitrospira
SU-WWTP	3	TN_R-	-0.5174	-0.3210	Unidentified
SU-WWTP	3	TN_R-	-0.5165	-0.5029	Unidentified
SU-WWTP	3	TP_R-	-0.4775	-0.5155	Unidentified
SU-WWTP	3	TP_R-		-0.5154	Unidentified
SU-WWTP	3	TP_R-	-0.4999	-0.5143	Kouleothrix
SU-WWTP	3	TP_R-		-0.5129	Unidentified
SU-WWTP	3	TN_R-	-0.5117	-0.4347	Unidentified
SU-WWTP	3	TP_R-	-0.4381	-0.5109	Turneriella
SU-WWTP	3	TP_R-	-0.3979	-0.5105	Unidentified
SU-WWTP	3	TN_R-	-0.5079	-0.4533	Unidentified
SU-WWTP	3	TP_R-	-0.4826	-0.5065	Ca_Sarcinithrix
SU-WWTP	3	TN_R-	-0.5052	-0.3726	Unidentified
SU-WWTP	3	TP_R-	-0.4276	-0.5052	Novosphingobium
SU-WWTP	3	TP_R-	-0.3369	-0.5045	Hirschia
SU-WWTP	3	TP_R-	-0.3962	-0.5039	Unidentified
SU-WWTP	3	TN_R-	-0.5033	-0.3509	Unidentified
SU-WWTP	3	TN_R-	-0.5028	-0.4703	Ferruginibacter
SU-WWTP	3	TN_R-	-0.5027	-0.3923	Unidentified

SU-WWTP	3	TP_R-	-0.3543	-0.5011	Runella
SU-WWTP	3	TP_R-	-0.4919	-0.5010	Unidentified
SU-WWTP	3	TP_R-	-0.4677	-0.4969	Unidentified
SU-WWTP	3	TP_R-	-0.3928	-0.4958	Fimbriiglobus
SU-WWTP	3	TP_R-	-0.3783	-0.4956	Unidentified
SU-WWTP	3	TP_R-	-0.3907	-0.4952	Unidentified
SU-WWTP	3	TP_R-	-0.4385	-0.4948	Unidentified
SU-WWTP	3	TP_R-		-0.4947	Terrimonas
SU-WWTP	3	TP_R-	-0.3898	-0.4941	Unidentified
SU-WWTP	3	TP_R-		-0.4937	Unidentified
SU-WWTP	3	TP_R-	-0.4657	-0.4927	Unidentified
SU-WWTP	3	TN_R-	-0.4909	-0.3169	Subgroup_10
SU-WWTP	3	TP_R-	-0.4415	-0.4907	Unidentified
SU-WWTP	3	TP_R-	-0.4733	-0.4906	Unidentified
SU-WWTP	3	TN_R-	-0.4906	-0.3504	Dechloromonas
SU-WWTP	3	TP_R-	-0.3925	-0.4899	Unidentified
SU-WWTP	3	TP_R-	-0.4271	-0.4879	Unidentified
SU-WWTP	3	TP_R-	-0.4632	-0.4877	Ca_Promineofilum
SU-WWTP	3	TN_R-	-0.4855	-0.4808	Unidentified
SU-WWTP	3	TP_R-		-0.4844	Haliangium
SU-WWTP	3	TN_R-	-0.4821		Crenothrix
SU-WWTP	2	TN_R-	-0.4820		Unidentified
SU-WWTP	3	TN_R-	-0.4816	-0.3557	Unidentified
SU-WWTP	3	TP_R-	-0.3240	-0.4811	Unidentified
SU-WWTP	3	TN_R-	-0.4795	-0.4524	Unidentified
SU-WWTP	3	TP_R-	-0.4239	-0.4772	Unidentified
SU-WWTP	3	TP_R-	-0.4291	-0.4762	Unidentified
SU-WWTP	3	TP_R-	-0.4161	-0.4761	Unidentified
SU-WWTP	3	TP_R-	-0.4305	-0.4760	Unidentified
SU-WWTP	3	TP_R-	-0.4545	-0.4749	Kouleothrix
SU-WWTP	3	TN_R-	-0.4736	-0.4541	Lentimicrobium
SU-WWTP	3	TP_R-		-0.4710	CL500-3
SU-WWTP	3	TN_R-	-0.4706	-0.3563	Unidentified
SU-WWTP	3	TN_R-	-0.4695	-0.4377	Unidentified
SU-WWTP	3	TN_R-	-0.4686	-0.4231	Ferruginibacter
SU-WWTP	3	TP_R-		-0.4674	Unidentified
SU-WWTP	3	TN_R-	-0.4669	-0.3859	Comamonas
SU-WWTP	2	TN_R-	-0.4656		Unidentified
SU-WWTP	3	TN_R-	-0.4655	-0.4285	Crenothrix
SU-WWTP	3	TP_R-	-0.3477	-0.4655	Iamia
SU-WWTP	3	TP_R-		-0.4654	Ca_Microthrix
SU-WWTP	3	TN_R-	-0.4651	-0.3600	Escherichia-Shigella
SU-WWTP	3	TP_R-	-0.4235	-0.4647	Unidentified
SU-WWTP	3	TP_R-	-0.4134	-0.4632	Phaselicystis
SU-WWTP	3	TP_R-	-0.4538	-0.4621	Unidentified
SU-WWTP	3	TN_R-	-0.4619	-0.4313	Unidentified

SU-WWTP	3	TP_R-		-0.4057	-0.4602	Unidentified
SU-WWTP	3	TN_R-		-0.4599	-0.4565	Unidentified
SU-WWTP	3	TP_R-		-0.3166	-0.4597	Tetrasphaera
SU-WWTP	3	TP_R-			-0.4597	JGI_0001001-H03
SU-WWTP	3	TP_R-		-0.3503	-0.4595	Unidentified
SU-WWTP	3	TN_R-		-0.4573		Dechloromonas
SU-WWTP	3	TN_R-		-0.4550	-0.4429	Unidentified
SU-WWTP	3	TN_R-		-0.4548	-0.4230	Unidentified
SU-WWTP	3	TP_R-		-0.3701	-0.4534	Unidentified
SU-WWTP	3	TP_R-			-0.4526	Unidentified
SU-WWTP	3	TN_R-		-0.4488	-0.3606	Unidentified
SU-WWTP	3	TN_R-		-0.4487	-0.3885	Ca_Promineofilum
SU-WWTP	3	TN_R-		-0.4471		Thiothrix
SU-WWTP	3	TP_R-		-0.3268	-0.4469	Kouleothrix
SU-WWTP	3	TN_R-		-0.4461		Unidentified
SU-WWTP	3	TP_R-			-0.4432	Hyphomicrobium
SU-WWTP	3	TP_R-		-0.4128	-0.4432	Unidentified
SU-WWTP	3	TN_R-		-0.4424		Unidentified
SU-WWTP	3	TP_R-			-0.4413	Unidentified
SU-WWTP	3	TN_R-		-0.4401	-0.3835	Unidentified
SU-WWTP	3	TN_R-		-0.4393	-0.3502	Unidentified
SU-WWTP	3	TP_R-		-0.4172	-0.4382	Unidentified
SU-WWTP	3	TP_R-			-0.4367	Lautropia
SU-WWTP	3	TP_R-		-0.3998	-0.4367	Kouleothrix
SU-WWTP	3	TP_R-		-0.4333	-0.4364	Unidentified
SU-WWTP	3	BOD_R-	-0.4362	-0.3096		Unidentified
SU-WWTP	3	TP_R-			-0.4362	Kouleothrix
SU-WWTP	3	TP_R-		-0.4298	-0.4357	Unidentified
SU-WWTP	3	TP_R-			-0.4347	Unidentified
SU-WWTP	3	TN_R-		-0.4346	-0.3427	Unidentified
SU-WWTP	3	TP_R-		-0.3776	-0.4331	Unidentified
SU-WWTP	3	TN_R-		-0.4310	-0.4191	Unidentified
SU-WWTP	3	TP_R-		-0.3738	-0.4308	Unidentified
SU-WWTP	3	TP_R-		-0.3938	-0.4293	Unidentified
SU-WWTP	3	TN_R-		-0.4288		Unidentified
SU-WWTP	3	TN_R-		-0.4280		Unidentified
SU-WWTP	3	TN_R-		-0.4276		SM1A02
SU-WWTP	3	TN_R-		-0.4276	-0.3227	Chitinivorax
SU-WWTP	2	TN_R-		-0.4268		Unidentified
SU-WWTP	3	TN_R-		-0.4259	-0.4184	Nitrosomonas
SU-WWTP	3	TP_R-		-0.3881	-0.4254	Arenimonas
SU-WWTP	3	TN_R-		-0.4244	-0.3270	Unidentified
SU-WWTP	3	TP_R-		-0.3890	-0.4240	Pir4_lineage
SU-WWTP	3	TP_R-		-0.3793	-0.4234	Unidentified
SU-WWTP	3	TN_R-		-0.4228	-0.4043	Unidentified
SU-WWTP	3	TN_R-		-0.4222	-0.3931	Nordella

SU-WWTP	3	TP_R-		-0.4222	JGI_0001001-H03
SU-WWTP	2	TN_R-	-0.4216	-0.3461	Unidentified
SU-WWTP	3	TP_R-		-0.4213	Kouleothrix
SU-WWTP	3	TP_R-		-0.4198	CL500-29_marine_group
SU-WWTP	3	TP_R-	-0.3136	-0.4195	Ellin6067
SU-WWTP	3	TN_R-	-0.4194	-0.3120	Dechloromonas
SU-WWTP	3	TP_R-	-0.3410	-0.4186	Unidentified
SU-WWTP	3	TP_R-		-0.4178	Oikopleura
SU-WWTP	3	TP_R-	-0.3991	-0.4167	Unidentified
SU-WWTP	3	TN_R-	-0.4148	-0.3075	Ca_Epiflobacter
SU-WWTP	3	TN_R-	-0.4147		Unidentified
SU-WWTP	3	TP_R-	-0.3200	-0.4145	Unidentified
SU-WWTP	3	TN_R-	-0.4129	-0.3801	Unidentified
SU-WWTP	3	TN_R-	-0.4128		SWB02
SU-WWTP	3	TP_R-		-0.4122	Unidentified
SU-WWTP	3	TN_R-	-0.4114		Dechloromonas
SU-WWTP	3	TN_R-	-0.4113		Unidentified
SU-WWTP	3	TN_R-	-0.4111		Unidentified
SU-WWTP	3	TP_R-	-0.3957	-0.4108	Ca_Alysiosphaera
SU-WWTP	3	TN_R-	-0.4099	-0.3030	Unidentified
SU-WWTP	3	TN_R-	-0.4094	-0.4025	Unidentified
SU-WWTP	3	TN_R-	-0.4093	-0.3305	Unidentified
SU-WWTP	3	TN_R-	-0.4092	-0.3226	Unidentified
SU-WWTP	3	TP_R-	-0.3979	-0.4085	Unidentified
SU-WWTP	3	TN_R-	-0.4075	-0.3422	Ca_Competibacter
SU-WWTP	2	TN_R-	-0.4065		Pirellula
SU-WWTP	3	TP_R-		-0.4064	Unidentified
SU-WWTP	3	TN_R-	-0.4063	-0.3462	Unidentified
SU-WWTP	3	TN_R-	-0.4061		Mycobacterium
SU-WWTP	2	TN_R-	-0.4061		Dechloromonas
SU-WWTP	3	TP_R-	-0.3340	-0.4060	SH-PL14
SU-WWTP	3	TN_R-	-0.4046		Unidentified
SU-WWTP	3	TP_R-	-0.3620	-0.4043	SM1A02
SU-WWTP	3	TN_R-	-0.4042		Unidentified
SU-WWTP	3	TN_R-	-0.4041	-0.3197	AKYG587
SU-WWTP	3	TP_R-	-0.3971	-0.4016	Unidentified
SU-WWTP	3	TN_R-	-0.4007	-0.3993	Ca_Megaira
SU-WWTP	3	TN_R-	-0.3996	-0.3871	Unidentified
SU-WWTP	3	TP_R-	-0.3953	-0.3992	Methylosarcina
SU-WWTP	3	TN_R-	-0.3989	-0.3260	Terrimonas
SU-WWTP	3	TN_R-	-0.3980		Unidentified
SU-WWTP	3	TP_R-		-0.3974	Neochlamydia
SU-WWTP	3	TP_R-	-0.3388	-0.3974	Unidentified
SU-WWTP	3	TP_R-		-0.3968	Unidentified
SU-WWTP	3	TP_R-		-0.3966	Methanothrix
SU-WWTP	3	TP_R-	-0.3317	-0.3961	Unidentified

SU-WWTP	3	TN_R-	-0.3958	-0.3791	Haliangium
SU-WWTP	3	TP_R-		-0.3957	Pirellula
SU-WWTP	2	TN_R-	-0.3953		Unidentified
SU-WWTP	3	TP_R-		-0.3952	Unidentified
SU-WWTP	3	TP_R-	-0.3848	-0.3936	Unidentified
SU-WWTP	3	TN_R-	-0.3931	-0.3129	Dechloromonas
SU-WWTP	3	TP_R-	-0.3336	-0.3929	Gemmata
SU-WWTP	3	TP_R-	-0.3092	-0.3920	Unidentified
SU-WWTP	2	TN_R-	-0.3920		Singulisphaera
SU-WWTP	3	TN_R-	-0.3919	-0.3056	Unidentified
SU-WWTP	3	TP_R-	-0.3656	-0.3918	Unidentified
SU-WWTP	3	TP_R-	-0.3639	-0.3917	Unidentified
SU-WWTP	3	TP_R-		-0.3915	Unidentified
SU-WWTP	3	TN_R-	-0.3909	-0.3878	Unidentified
SU-WWTP	2	TN_R-	-0.3905	-0.3338	Unidentified
SU-WWTP	3	TN_R-	-0.3904	-0.3613	Sphingopyxis
SU-WWTP	3	TP_R-		-0.3902	Unidentified
SU-WWTP	3	TP_R-		-0.3900	Unidentified
SU-WWTP	3	TN_R-	-0.3896		Unidentified
SU-WWTP	2	TN_R-	-0.3889		Unidentified
SU-WWTP	3	TN_R-	-0.3885	-0.3328	Kouleothrix
SU-WWTP	3	TN_R-	-0.3884	-0.3198	Unidentified
SU-WWTP	2	TN_R-	-0.3880		Unidentified
SU-WWTP	3	TP_R-	-0.3218	-0.3878	Stella
SU-WWTP	3	TP_R-		-0.3875	Unidentified
SU-WWTP	3	TP_R-		-0.3873	Unidentified
SU-WWTP	3	TP_R-		-0.3868	Unidentified
SU-WWTP	2	TN_R-	-0.3865		Unidentified
SU-WWTP	3	TP_R-		-0.3861	Stenotrophobacter
SU-WWTP	3	TN_R-	-0.3846	-0.3516	OM27_clade
SU-WWTP	3	TP_R-		-0.3843	Ferruginibacter
SU-WWTP	3	TP_R-	-0.3595	-0.3839	Haliangium
SU-WWTP	3	TN_R-	-0.3836	-0.3428	Unidentified
SU-WWTP	3	TN_R-	-0.3835	-0.3489	Sandaracinus
SU-WWTP	3	TP_R-		-0.3823	OLB8
SU-WWTP	3	TP_R-		-0.3819	Ferruginibacter
SU-WWTP	3	TP_R-		-0.3815	Pirellula
SU-WWTP	3	TN_R-	-0.3805	-0.3480	Neochlamydia
SU-WWTP	3	TP_R-		-0.3792	Unidentified
SU-WWTP	3	TN_R-	-0.3790		Unidentified
SU-WWTP	3	TN_R-	-0.3780		Unidentified
SU-WWTP	3	TP_R-	-0.3635	-0.3774	Unidentified
SU-WWTP	3	TN_R-	-0.3773	-0.3183	Agitococcus_lubricus_group
SU-WWTP	3	TN_R-	-0.3765		Pajaroellobacter
SU-WWTP	3	TP_R-		-0.3762	Blastocatella
SU-WWTP	3	TP_R-		-0.3755	Ideonella

SU-WWTP	3	TN_R-	-0.3754	-0.3327	Blastopirellula
SU-WWTP	3	TN_R-	-0.3742	-0.3415	Nannocystis
SU-WWTP	3	TP_R-	-0.3310	-0.3735	Chthoniobacter
SU-WWTP	3	TP_R-	-0.3205	-0.3717	Unidentified
SU-WWTP	3	TP_R-		-0.3715	OLB12
SU-WWTP	3	TN_R-	-0.3714		Pirellula
SU-WWTP	3	TN_R-	-0.3709	-0.3032	Pirellula
SU-WWTP	3	TP_R-		-0.3708	Unidentified
SU-WWTP	3	TN_R-	-0.3704		SH3-11
SU-WWTP	3	TP_R-		-0.3704	Nannocystis
SU-WWTP	3	TP_R-	-0.3527	-0.3699	Gordonia
SU-WWTP	3	TP_R-		-0.3690	Unidentified
SU-WWTP	3	TP_R-		-0.3688	Unidentified
SU-WWTP	3	TP_R-		-0.3681	Unidentified
SU-WWTP	3	TP_R-		-0.3676	Flavobacterium
SU-WWTP	3	TP_R-		-0.3673	Unidentified
SU-WWTP	3	TP_R-		-0.3662	Unidentified
SU-WWTP	3	TN_R-	-0.3653		Oikopleura
SU-WWTP	3	TN_R-	-0.3651	-0.3167	Bradyrhizobium
SU-WWTP	3	TP_R-		-0.3645	Thermomonas
SU-WWTP	3	TN_R-	-0.3644	-0.3632	Tetrasphaera
SU-WWTP	3	TP_R-		-0.3641	Unidentified
SU-WWTP	3	TN_R-	-0.3635		Reyranella
SU-WWTP	3	TP_R-		-0.3634	Unidentified
SU-WWTP	3	TN_R-	-0.3628	-0.3142	Unidentified
SU-WWTP	3	TN_R-	-0.3620		Unidentified
SU-WWTP	3	TN_R-	-0.3615		Halomonas
SU-WWTP	3	TP_R-		-0.3614	Unidentified
SU-WWTP	3	TP_R-	-0.3128	-0.3613	Unidentified
SU-WWTP	3	TP_R-		-0.3611	Terrimonas
SU-WWTP	3	TP_R-		-0.3605	Polymorphobacter
SU-WWTP	2	TN_R-	-0.3588		Pseudomonas
SU-WWTP	3	TP_R-	-0.3200	-0.3586	Unidentified
SU-WWTP	3	TP_R-		-0.3586	Unidentified
SU-WWTP	3	TP_R-		-0.3582	Unidentified
SU-WWTP	3	TP_R-		-0.3575	Unidentified
SU-WWTP	3	TP_R-		-0.3572	Unidentified
SU-WWTP	3	TP_R-		-0.3570	Unidentified
SU-WWTP	3	TP_R-	-0.3059	-0.3563	Unidentified
SU-WWTP	3	TP_R-		-0.3551	Prevotella_9
SU-WWTP	3	TN_R-	-0.3530		Turneriella
SU-WWTP	3	TP_R-	-0.3297	-0.3529	Unidentified
SU-WWTP	3	TN_R-	-0.3528		Lentimicrobium
SU-WWTP	3	TP_R-	-0.3332	-0.3516	Unidentified
SU-WWTP	3	TP_R-		-0.3509	SM1A02
SU-WWTP	3	TP_R-		-0.3507	Unidentified

SU-WWTP	3	TN_R-	-0.3507		Unidentified
SU-WWTP	3	TP_R-		-0.3501	Marmoricola
SU-WWTP	1	TN_R+	0.3504	0.3289	Unidentified
SU-WWTP	2	TP_R+		0.3512	Sphaerotilus
SU-WWTP	1	TP_R+		0.3514	Pirellula
SU-WWTP	2	TN_R+	0.3535	0.3015	Unidentified
SU-WWTP	1	TP_R+		0.3544	Ferruginibacter
SU-WWTP	1	TN_R+	0.3547		Unidentified
SU-WWTP	1	TP_R+		0.3557	Pirellula
SU-WWTP	1	TP_R+		0.3574	Unidentified
SU-WWTP	1	TN_R+	0.3583		Verrucomicrobium
SU-WWTP	1	TP_R+		0.3609	Unidentified
SU-WWTP	1	TP_R+		0.3616	Unidentified
SU-WWTP	3	TN_R+	0.3617		XBB1006
SU-WWTP	1	TN_R+	0.3621		Unidentified
SU-WWTP	2	TN_R+	0.3639		Rhodobacter
SU-WWTP	1	TP_R+	0.3057	0.3643	Unidentified
SU-WWTP	1	TN_R+	0.3713	0.3685	Unidentified
SU-WWTP	1	TP_R+		0.3721	Unidentified
SU-WWTP	1	TP_R+		0.3747	Unidentified
SU-WWTP	3	TN_R+	0.3759		Ramlibacter
SU-WWTP	1	TP_R+	0.3605	0.3770	Bradyrhizobium
SU-WWTP	1	TP_R+		0.3772	Unidentified
SU-WWTP	2	TP_R+		0.3792	Gordonia
SU-WWTP	2	TN_R+	0.3832	0.3641	Streptococcus
SU-WWTP	1	TP_R+	0.3626	0.3851	Unidentified
SU-WWTP	3	TN_R+	0.3879		Ca_Accumulibacter
SU-WWTP	1	TP_R+		0.3898	Unidentified
SU-WWTP	1	TN_R+	0.3899	0.3624	Unidentified
SU-WWTP	1	TN_R+	0.3906	0.3497	Unidentified
SU-WWTP	1	TN_R+	0.3916	0.3822	CL500-29_marine_group
SU-WWTP	1	TN_R+	0.3921	0.3866	Unidentified
SU-WWTP	1	TN_R+	0.3931	0.3584	Unidentified
SU-WWTP	1	TP_R+		0.3965	Anaerolinea
SU-WWTP	1	TP_R+	0.3156	0.4023	Subgroup_10
SU-WWTP	2	TP_R+		0.4048	Ca_Promineofilum
SU-WWTP	3	TP_R+	0.3664	0.4058	Unidentified
SU-WWTP	2	TP_R+		0.4062	Leptolinea
SU-WWTP	1	TP_R+		0.4069	Unidentified
SU-WWTP	1	TN_R+	0.4089		Tetrasphaera
SU-WWTP	2	TN_R+	0.4144		Bdellovibrio
SU-WWTP	1	TP_R+	0.4125	0.4182	Ellin6067
SU-WWTP	1	TP_R+	0.3270	0.4192	Unidentified
SU-WWTP	1	TP_R+	0.3332	0.4204	Ca_Amarolinea
SU-WWTP	2	TP_R+	0.3398	0.4208	Unidentified
SU-WWTP	1	TN_R+	0.4244	0.3130	Unidentified

SU-WWTP	1	TN_R+		0.4252	0.3318	Unidentified
SU-WWTP	2	TP_R+		0.3053	0.4253	Ca_Methylophosphatis
SU-WWTP	1	TP_R+		0.3964	0.4255	Unidentified
SU-WWTP	3	TN_R+		0.4255		Actinomyces
SU-WWTP	2	TN_R+		0.4283		Enhydrobacter
SU-WWTP	2	TN_R+		0.4309	0.3478	Unidentified
SU-WWTP	2	TP_R+		0.3436	0.4322	Unidentified
SU-WWTP	1	TP_R+		0.3691	0.4342	Unidentified
SU-WWTP	1	TP_R+		0.3779	0.4356	Unidentified
SU-WWTP	1	TP_R+			0.4374	BD1-7_clade
SU-WWTP	1	TN_R+		0.4391	0.3879	Leptothrix
SU-WWTP	2	TP_R+			0.4434	Unidentified
SU-WWTP	1	TP_R+			0.4445	Unidentified
SU-WWTP	1	TP_R+			0.4468	Geothrix
SU-WWTP	3	BOD_R+	0.4507			Unidentified
SU-WWTP	1	TP_R+		0.3916	0.4524	Unidentified
SU-WWTP	1	TN_R+		0.4525	0.4080	Terrimonas
SU-WWTP	2	TN_R+		0.4527		Ruminococcus
SU-WWTP	3	TN_R+		0.4572	0.4528	Unidentified
SU-WWTP	1	TP_R+		0.3927	0.4585	Unidentified
SU-WWTP	1	TP_R+			0.4606	SM1A02
SU-WWTP	2	TP_R+		0.4463	0.4634	Unidentified
SU-WWTP	1	TP_R+		0.3495	0.4646	Unidentified
SU-WWTP	3	TN_R+		0.4672	0.3854	Unidentified
SU-WWTP	2	BOD_R+	0.4739			Ca_Epiflobacter
SU-WWTP	1	TP_R+		0.4371	0.4765	Unidentified
SU-WWTP	1	TP_R+		0.4236	0.4780	Unidentified
SU-WWTP	1	TP_R+		0.3829	0.4798	Fimbriiglobus
SU-WWTP	1	TN_R+		0.4812	0.4122	Terrimonas
SU-WWTP	1	TP_R+		0.3969	0.4849	Unidentified
SU-WWTP	1	TP_R+		0.4642	0.4857	Fimbriiglobus
SU-WWTP	1	TP_R+		0.3795	0.4868	Unidentified
SU-WWTP	1	TN_R+		0.4900	0.4734	Unidentified
SU-WWTP	1	TP_R+		0.3109	0.4908	Unidentified
SU-WWTP	1	TN_R+		0.4970	0.4230	Unidentified
SU-WWTP	1	TP_R+		0.4087	0.4980	Unidentified
SU-WWTP	1	TP_R+		0.4244	0.4985	JGI_0001001-H03
SU-WWTP	1	TN_R+		0.4997	0.4277	Unidentified
SU-WWTP	1	TP_R+		0.4247	0.5009	Unidentified
SU-WWTP	1	TP_R+		0.3959	0.5072	Unidentified
SU-WWTP	1	TP_R+		0.4771	0.5114	Unidentified
SU-WWTP	3	TN_R+		0.5196	0.3933	Lacihabitans
SU-WWTP	1	TN_R+		0.5215	0.4497	Marmoricola
SU-WWTP	1	TP_R+		0.4666	0.5270	Prosthecobacter
SU-WWTP	1	TP_R+			0.5286	Unidentified
SU-WWTP	1	TN_R+		0.5307	0.5274	Unidentified

SU-WWTP		1	TN_R+	0.5323	0.5015	Unidentified
SU-WWTP		1	TP_R+	0.5049	0.5371	Nitrosomonas
SU-WWTP		1	TP_R+	0.4026	0.5377	Ca_Sarcinithrix
SU-WWTP		1	TP_R+	0.4326	0.5382	Unidentified
SU-WWTP		1	TN_R+	0.5415	0.4139	Unidentified
SU-WWTP		1	TP_R+	0.4393	0.5534	Ca_Amarolinea
SU-WWTP		1	TN_R+	0.5540	0.5393	Unidentified
SU-WWTP		1	TP_R+	0.4516	0.5591	SH-PL14
SU-WWTP		1	TN_R+	0.5605	0.5213	Schlesneria
SU-WWTP		1	TP_R+		0.5636	Unidentified
SU-WWTP		1	TP_R+	0.5033	0.5675	Unidentified
SU-WWTP		1	TP_R+	0.5344	0.5703	Ahniella
SU-WWTP		1	TP_R+	0.4354	0.5724	Unidentified
SU-WWTP		1	TP_R+	0.4787	0.5737	Uliginosibacterium
SU-WWTP		1	TP_R+	0.5068	0.5748	Bryobacter
SU-WWTP		1	TN_R+	0.5748	0.5029	Unidentified
SU-WWTP		1	TN_R+	0.5832	0.5785	Mesorhizobium
SU-WWTP		1	TN_R+	0.5866	0.5695	Unidentified
SU-WWTP		1	TP_R+	0.5104	0.6085	Unidentified
SU-WWTP		1	TP_R+	0.4937	0.6085	Unidentified
SU-WWTP		1	TP_R+	0.4841	0.6123	Unidentified
SU-WWTP		1	TP_R+	0.5278	0.6129	Unidentified
SU-WWTP		1	TP_R+	0.5139	0.6159	Unidentified
SU-WWTP		1	TP_R+	0.5770	0.6170	Unidentified
SU-WWTP		1	TP_R+	0.5113	0.6258	Unidentified
SU-WWTP		1	TP_R+	0.4612	0.6395	Unidentified
SU-WWTP		1	TP_R+	0.5465	0.6414	Unidentified
SU-WWTP		1	TP_R+	0.5677	0.6441	Unidentified
SU-WWTP		1	TP_R+	0.5429	0.6673	Unidentified
SU-WWTP		1	TP_R+	0.6286	0.6697	Ahniella
SU-WWTP		1	TP_R+	0.6307	0.6757	Unidentified
SU-WWTP		1	TP_R+	0.5870	0.6901	Unidentified
SU-WWTP		1	TP_R+	0.6955	0.7008	JGI_0001001-H03

SUPPLEMENTARY TABLE 2. Spearman's rank correlation coefficients between time-point network topology metrics. On the left coefficients of FF-WWTP and on the right coefficients of SU-WWTP.

	Modularity	Clustering coefficient	Porportion of co-exclusions
Modularity	1/1	-0.50/-0.87	0.52/0.85
Clustering coefficient	-0.50/-0.87	1/1	-0.58/-0.87
Porportion of co-exclusions	0.52/0.85	-0.58/-0.87	1/1

SUPPLEMENTARY TABLE 3. Phylogenetic dispersion and α-diversity metrics

Sample	WWTP	NTI	NRI	^{0}pD	^{0}D	^{1}D	^{2}D
FF.1	FF-WWTP	2,916	2,740	184,506	1473	388,696	149,829
FF.1	FF-WWTP	3,528	3,100	116,099	850	297,363	128,897
FF.1	FF-WWTP	3,967	2,134	172,512	1520	538,378	192,794
FF.2	FF-WWTP	4,026	2,580	88,839	621	282,104	138,860
FF.2	FF-WWTP	3,337	2,308	98,107	584	273,897	128,528
FF.2	FF-WWTP	3,358	2,435	100,254	737	304,304	136,959
FF.3	FF-WWTP	3,588	2,489	81,924	502	236,022	118,809
FF.3	FF-WWTP	3,280	2,058	86,399	540	248,731	122,555
FF.3	FF-WWTP	3,115	2,426	145,577	1048	365,504	142,765
FF.4	FF-WWTP	3,061	2,062	109,172	826	299,560	118,162
FF.4	FF-WWTP	3,221	2,165	88,644	594	253,308	117,671
FF.4	FF-WWTP	3,009	2,512	108,361	790	303,667	122,999
FF.5	FF-WWTP	1,082	-0,101	216,730	1402	302,354	90,122
FF.5	FF-WWTP	1,580	1,347	149,209	1019	237,873	55,414
FF.5	FF-WWTP	1,464	1,344	172,312	1109	259,117	58,257
FF.6	FF-WWTP	2,505	2,721	132,935	1182	333,558	98,334
FF.6	FF-WWTP	2,066	2,141	141,117	1230	321,288	90,394
FF.6	FF-WWTP	2,120	2,423	148,211	1312	356,721	104,740
FF.7	FF-WWTP	2,602	2,112	117,632	945	342,839	126,053
FF.7	FF-WWTP	2,451	1,755	102,621	779	282,682	90,290
FF.7	FF-WWTP	2,490	1,616	91,815	609	247,865	87,373
FF.8	FF-WWTP	2,422	2,458	162,517	1424	397,315	134,168
FF.8	FF-WWTP	2,515	2,334	159,900	1241	386,777	136,543
FF.8	FF-WWTP	2,428	1,892	128,203	885	300,311	104,709
FF.9	FF-WWTP	3,048	2,432	72,049	555	219,314	77,446
FF.9	FF-WWTP	2,604	2,338	90,772	785	246,826	76,412
FF.9	FF-WWTP	2,560	1,803	67,847	492	156,517	48,042
FF.10	FF-WWTP	2,883	1,822	69,411	414	118,352	42,964
FF.10	FF-WWTP	2,584	1,620	175,033	1404	342,288	109,255
FF.10	FF-WWTP	2,655	1,257	143,208	1023	309,508	102,315
FF.11	FF-WWTP	3,082	2,143	133,005	1132	357,864	139,242
FF.11	FF-WWTP	3,265	1,879	121,751	850	322,965	138,961
FF.11	FF-WWTP	3,448	2,216	92,784	688	257,914	108,333
FF.12	FF-WWTP	3,106	1,705	99,406	739	280,581	122,597
FF.12	FF-WWTP	3,128	1,437	91,962	631	254,705	113,531
FF.12	FF-WWTP	3,084	1,169	114,350	721	276,564	116,818
FF.13	FF-WWTP	3,784	1,963	98,862	736	306,322	146,062
FF.13	FF-WWTP	3,027	1,317	130,142	885	322,790	130,267
FF.13	FF-WWTP	3,159	2,178	147,219	1171	380,680	158,659
FF.14	FF-WWTP	2,614	1,688	120,419	910	294,452	103,379
FF.14	FF-WWTP	2,791	1,988	142,510	1017	344,048	125,267
FF.14	FF-WWTP	2,440	1,575	137,053	946	312,904	108,792
FF.15	FF-WWTP	2,515	1,282	121,119	746	266,865	98,580

FF.15	FF-WWTP	2,501	1,247	121,052	691	248,646	90,216
FF.15	FF-WWTP	2,729	1,367	115,189	645	243,615	93,836
FF.16	FF-WWTP	2,052	1,353	80,895	459	150,479	43,800
FF.16	FF-WWTP	2,092	1,434	114,474	753	243,894	72,382
FF.16	FF-WWTP	2,351	1,461	78,133	472	174,509	59,429
FF.17	FF-WWTP	1,917	1,522	123,171	777	237,160	65,287
FF.17	FF-WWTP	1,698	1,178	138,300	862	238,038	61,537
FF.17	FF-WWTP	1,758	1,210	103,957	578	167,757	42,114
FF.18	FF-WWTP	2,245	1,221	131,788	967	264,242	81,014
FF.18	FF-WWTP	2,161	1,237	143,036	1106	285,644	85,166
FF.18	FF-WWTP	2,060	1,146	144,395	1106	274,529	78,418
FF.19	FF-WWTP	2,066	1,884	163,994	1421	328,291	90,976
FF.19	FF-WWTP	2,123	1,881	163,551	1405	320,854	92,485
FF.19	FF-WWTP	2,238	1,698	101,527	724	218,038	60,478
FF.20	FF-WWTP	2,905	1,990	132,964	990	322,360	127,107
FF.20	FF-WWTP	2,534	1,873	134,991	1004	305,849	112,897
FF.20	FF-WWTP	2,581	1,977	140,904	1135	329,717	121,067
FF.21	FF-WWTP	2,889	1,229	61,702	344	134,378	59,687
FF.21	FF-WWTP	2,704	1,587	95,511	729	203,920	73,332
FF.21	FF-WWTP	2,887	1,232	62,652	354	137,590	58,296
FF.22	FF-WWTP	2,490	1,263	135,788	887	219,921	83,721
FF.22	FF-WWTP	2,416	1,370	151,786	1138	279,975	100,608
FF.22	FF-WWTP	2,662	1,455	143,368	1053	297,078	114,149
SU.1	SU-WWTP	4,111	-0,096	166,161	1254	493,825	219,903
SU.1	SU-WWTP	4,078	0,137	171,170	1240	507,614	228,183
SU.1	SU-WWTP	4,867	0,039	159,971	1271	546,528	262,128
SU.2	SU-WWTP	3,112	-0,219	180,279	1469	498,130	151,934
SU.2	SU-WWTP	2,853	-0,019	315,419	3679	742,965	190,802
SU.2	SU-WWTP	4,165	0,351	183,042	1460	565,686	215,854
SU.3	SU-WWTP	4,063	-0,870	94,026	582	302,404	135,486
SU.3	SU-WWTP	4,202	-0,909	111,928	716	359,346	162,555
SU.3	SU-WWTP	4,080	-0,837	75,255	408	238,534	126,593
SU.4	SU-WWTP	3,489	0,947	156,368	1239	538,666	217,571
SU.4	SU-WWTP	3,362	0,884	200,357	1816	649,229	236,013
SU.4	SU-WWTP	4,049	0,940	144,110	1139	520,889	222,652
SU.5	SU-WWTP	2,871	-0,747	196,046	1540	547,330	151,901
SU.5	SU-WWTP	3,849	-2,798	205,352	1769	750,927	235,240
SU.5	SU-WWTP	3,639	-0,199	161,345	1281	479,719	155,672
SU.6	SU-WWTP	3,768	0,339	228,565	2124	613,284	194,432
SU.6	SU-WWTP	3,789	0,562	211,127	1999	638,404	218,643
SU.6	SU-WWTP	3,645	0,533	145,140	1151	434,100	167,123
SU.7	SU-WWTP	4,541	0,515	162,600	1515	507,394	194,096
SU.7	SU-WWTP	3,487	-0,050	175,839	1551	493,093	168,510
SU.7	SU-WWTP	4,440	0,368	173,965	1746	479,637	156,768
SU.8	SU-WWTP	4,193	0,339	178,535	1600	537,268	200,770
SU.8	SU-WWTP	4,109	0,421	201,663	2124	622,142	221,291

SU.8	SU-WWTP	4,776	0,104	94,218	658	320,534	159,488
SU.9	SU-WWTP	4,684	0,376	160,835	1346	600,618	275,883
SU.9	SU-WWTP	5,384	0,885	154,965	1453	630,854	285,258
SU.9	SU-WWTP	5,709	0,506	69,870	460	273,922	158,474
SU.10	SU-WWTP	4,382	0,087	168,534	1433	593,177	272,635
SU.10	SU-WWTP	4,504	0,059	179,254	1626	644,769	292,732
SU.10	SU-WWTP	3,964	-0,045	174,781	1557	578,214	241,660
SU.11	SU-WWTP	4,118	-0,029	65,727	356	204,550	125,206
SU.11	SU-WWTP	4,520	0,930	122,359	988	421,188	202,738
SU.11	SU-WWTP	5,535	0,789	64,616	371	236,576	159,794
SU.12	SU-WWTP	6,072	0,185	116,262	859	420,845	235,538
SU.12	SU-WWTP	5,764	-0,016	103,361	718	372,696	214,905
SU.12	SU-WWTP	5,905	0,025	129,333	1039	476,180	251,474
SU.13	SU-WWTP	4,946	1,133	179,568	1310	538,846	203,140
SU.13	SU-WWTP	5,316	0,037	135,075	1010	455,724	225,388
SU.13	SU-WWTP	5,956	0,394	128,319	1041	481,344	251,929
SU.14	SU-WWTP	5,862	1,271	124,590	984	485,359	258,298
SU.14	SU-WWTP	5,829	1,246	141,079	1128	530,510	277,630
SU.14	SU-WWTP	5,400	1,302	147,768	1197	571,316	292,841
SU.15	SU-WWTP	5,103	0,763	146,934	1156	543,091	277,148
SU.15	SU-WWTP	5,125	0,842	149,516	1136	536,229	277,216
SU.15	SU-WWTP	5,030	0,992	141,773	1121	519,902	259,811
SU.17	SU-WWTP	3,852	0,445	125,456	875	354,914	99,179
SU.17	SU-WWTP	4,177	0,471	128,567	866	357,924	115,349
SU.17	SU-WWTP	3,660	0,534	138,168	947	361,295	98,668
SU.18	SU-WWTP	3,835	0,985	117,099	779	346,518	122,458
SU.18	SU-WWTP	3,802	0,206	135,732	978	368,083	93,790
SU.18	SU-WWTP	3,775	0,212	172,529	1248	434,822	115,537
SU.19	SU-WWTP	4,645	0,662	99,308	609	310,967	150,788
SU.19	SU-WWTP	4,243	0,021	100,275	604	306,169	131,803
SU.19	SU-WWTP	4,600	0,063	98,029	627	314,247	138,267
SU.20	SU-WWTP	4,240	0,265	120,333	738	371,586	173,826
SU.20	SU-WWTP	4,501	0,047	117,764	746	346,818	147,218
SU.20	SU-WWTP	5,112	-0,062	66,609	369	195,677	92,667
SU.21	SU-WWTP	5,027	0,648	83,179	492	271,925	134,163
SU.21	SU-WWTP	4,938	0,303	83,065	471	257,431	118,326
SU.21	SU-WWTP	4,563	0,588	57,894	292	185,059	101,492
SU.22	SU-WWTP	5,029	1,437	125,420	957	419,274	174,075
SU.22	SU-WWTP	5,245	1,052	78,596	450	252,847	125,708
SU.22	SU-WWTP	5,424	1,270	88,966	562	292,059	137,011
SU.23	SU-WWTP	4,939	0,742	84,170	489	248,537	102,820
SU.23	SU-WWTP	4,772	1,671	149,735	1367	471,067	152,003
SU.23	SU-WWTP	4,479	1,151	155,297	1426	463,894	139,326
SU.24	SU-WWTP	4,705	0,617	76,909	426	229,224	105,161
SU.24	SU-WWTP	4,802	0,693	72,060	396	216,381	98,833
SU.24	SU-WWTP	4,616	0,562	74,007	391	210,304	94,263

SUPPLEMENTARY DATA 1. Bray-Curtis dissimilarity matrix and probability of co-occurrence values for FF-WWTP and SU-WWTP (see separate file).

SUPPLEMENTARY DATA 2. Detailed information about sample characteristics, operational and environmental parameters, and time-point network properties (see separate file).

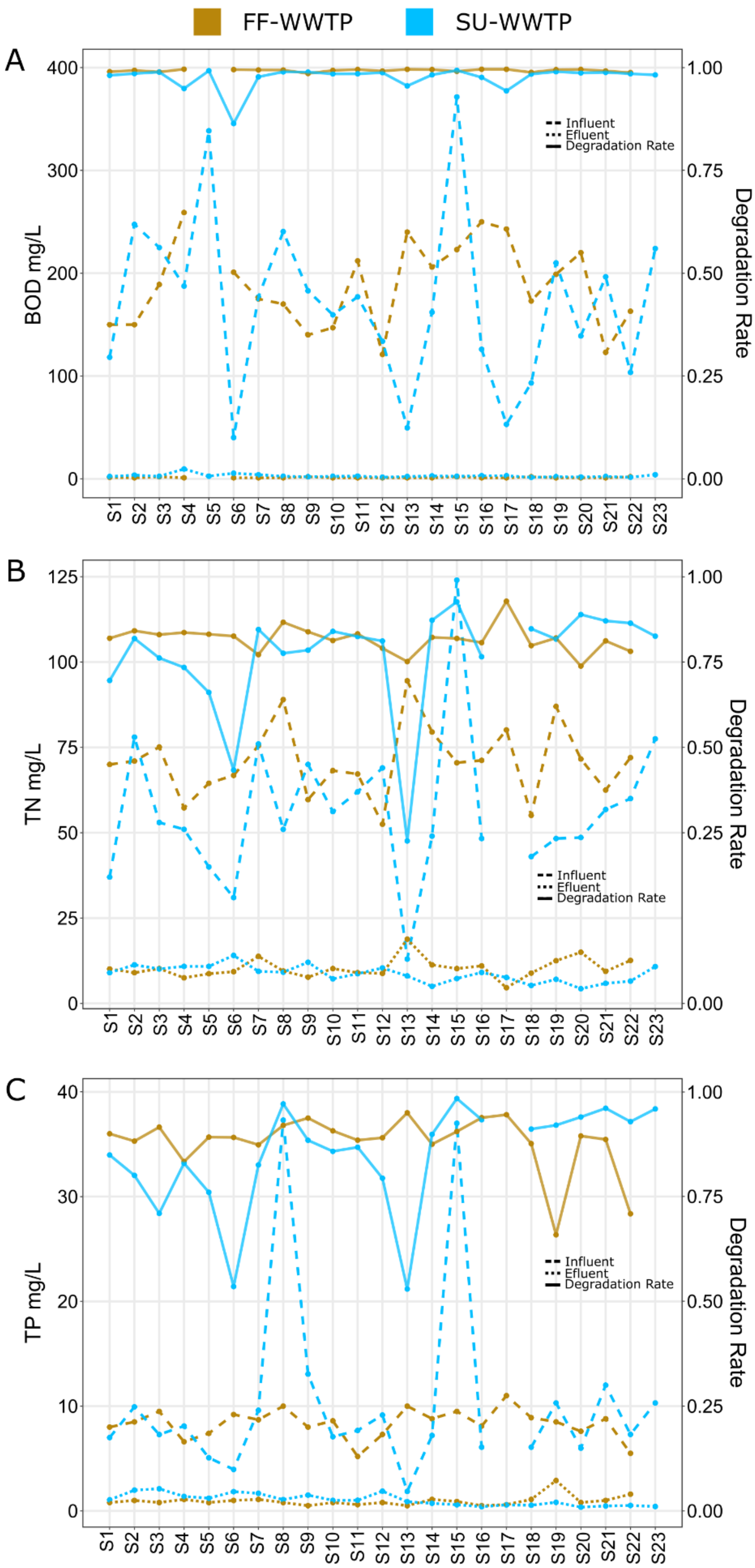

Supplementary Figure 1. Pollutant load. A) Biological oxygen demand. B) Total nitrogen. C) Total phosphorous. Smooth lines represent degradation rates, dashed lines concentrations at the influent and dotted lines concentrations at the effluent.

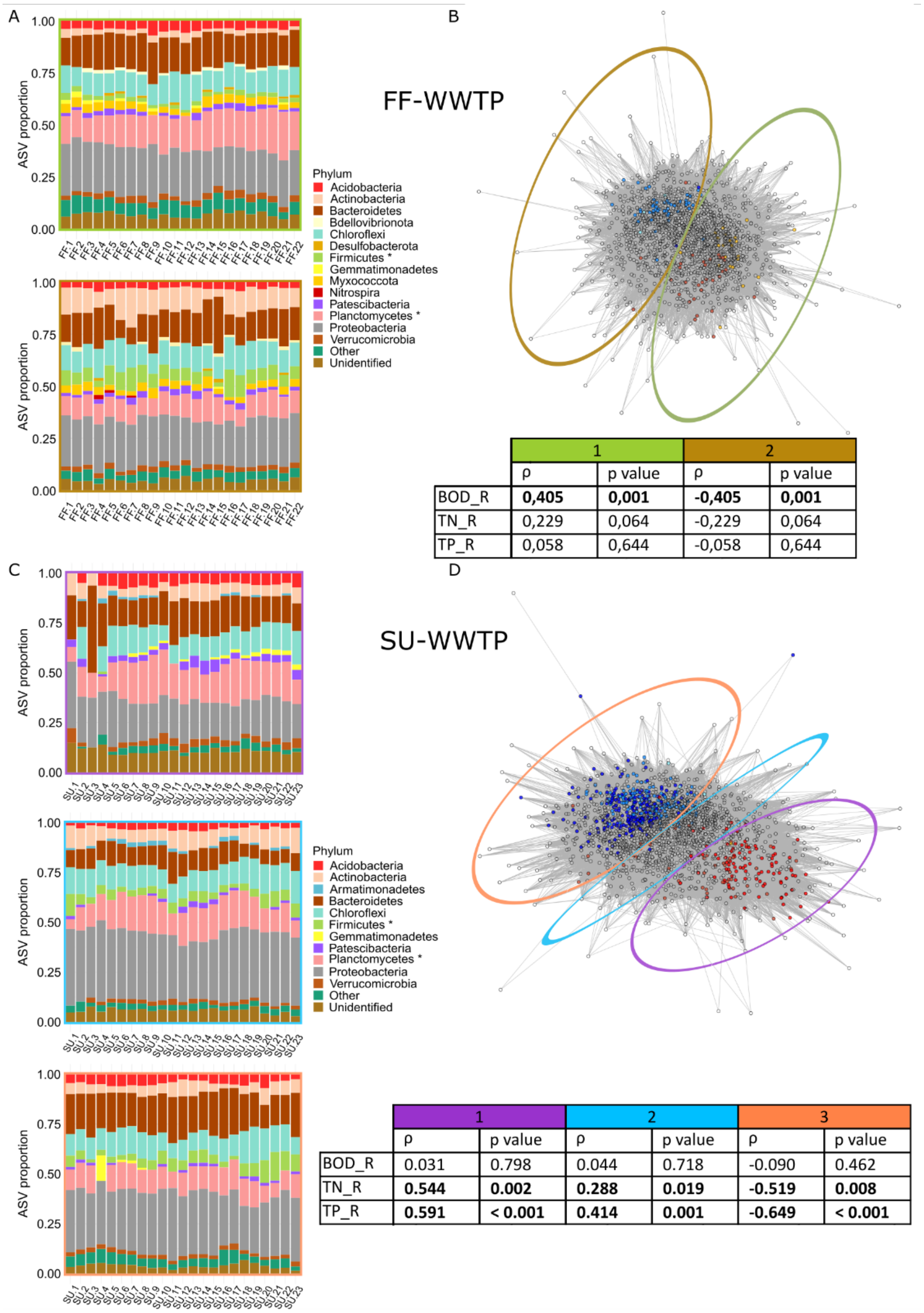

	1		2	
	ρ	p value	ρ	p value
BOD_R	**0,405**	**0,001**	**-0,405**	**0,001**
TN_R	0,229	0,064	-0,229	0,064
TP_R	0,058	0,644	-0,058	0,644

	1		2		3	
	ρ	p value	ρ	p value	ρ	p value
BOD_R	0.031	0.798	0.044	0.718	-0.090	0.462
TN_R	**0.544**	**0.002**	**0.288**	**0.019**	**-0.519**	**0.008**
TP_R	**0.591**	**< 0.001**	**0.414**	**0.001**	**-0.649**	**< 0.001**

Supplementary Figure 2. Taxonomic profiling al the phylum level of modules present in activated sludge communities. A) Modules present in FF-WWTP. B) FF-WWTP metaweb where nodes are colored by their main correlating pollutant degradation rate. C) Modules present in SU-WWTP. D) SU-WWTP metaweb where nodes are colored by their main correlating pollutant degradation rate. Firmicutes and Planctomycetes had significative differences in abundance in the different modules of the same WWTP (Student's t-test, $p < 0.05$). Tables represent the Spearman's rank correlations between module completeness and the pollutant degradation rates.

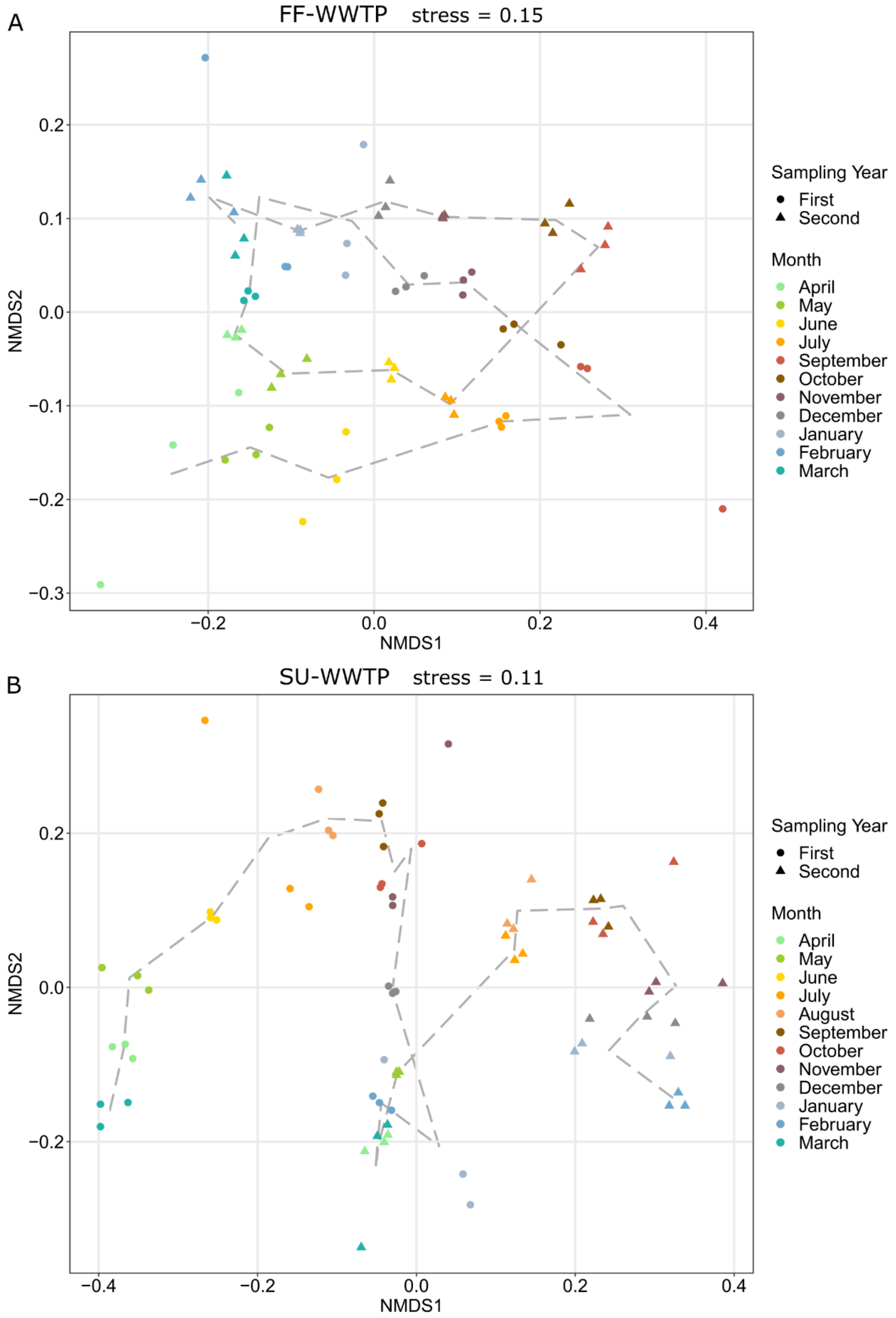

Supplementary Figure 3. Beta diversity ordinations of FF-WWTP and SU-WWTP microbial communities. Non-metric multidimensional scaling (nmds) calculated from Bray-Curtis dissimilarity matrices of ASV abundance data A) FF-WWTP and B) SU-WWTP. Dashed line shows the temporal succession of samples, showing the temporal dynamics of the microbial community.

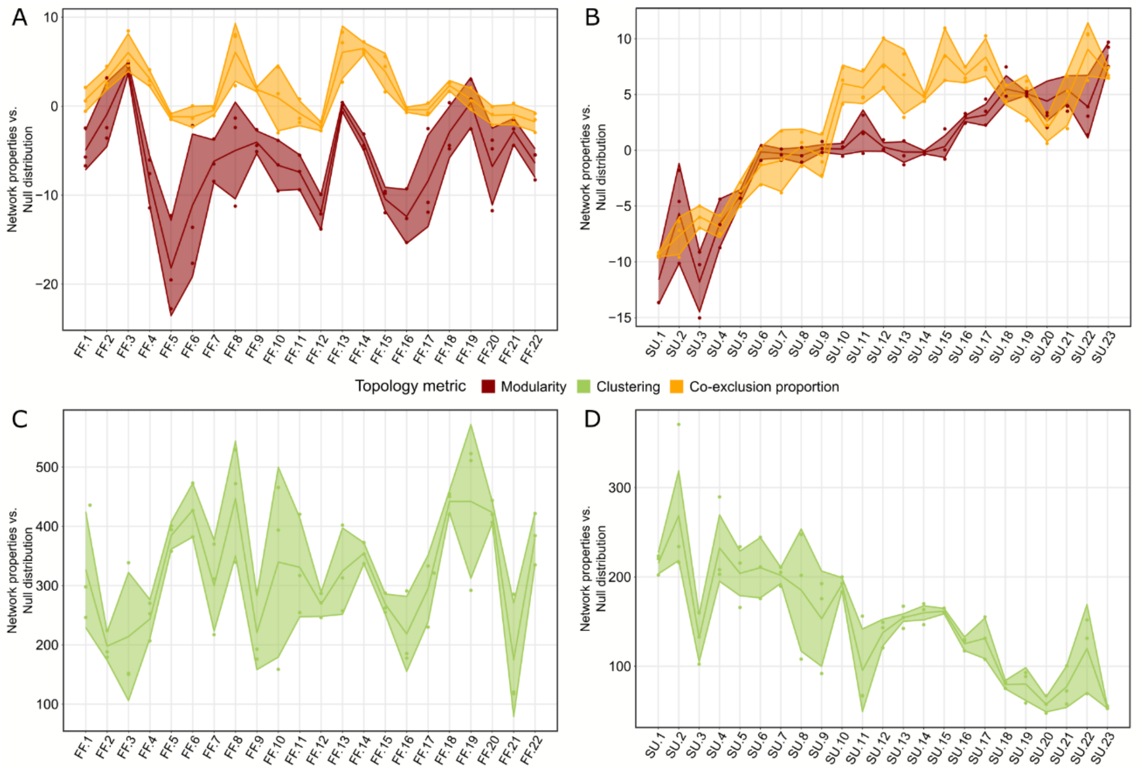

Supplementary Figure 4. Observed time-point network properties compared to null distributions. Modularity and co-exclusion proportion **A**) in the FF-WWTP **B**) and SU-WWTP. Clustering coefficient **C**) in the FF-WWTP **D**) and SU-WWTP. Dots represent sample data (n = 3), the central lines correspond to average values of each sample, and the shaded areas correspond to the standard deviation.

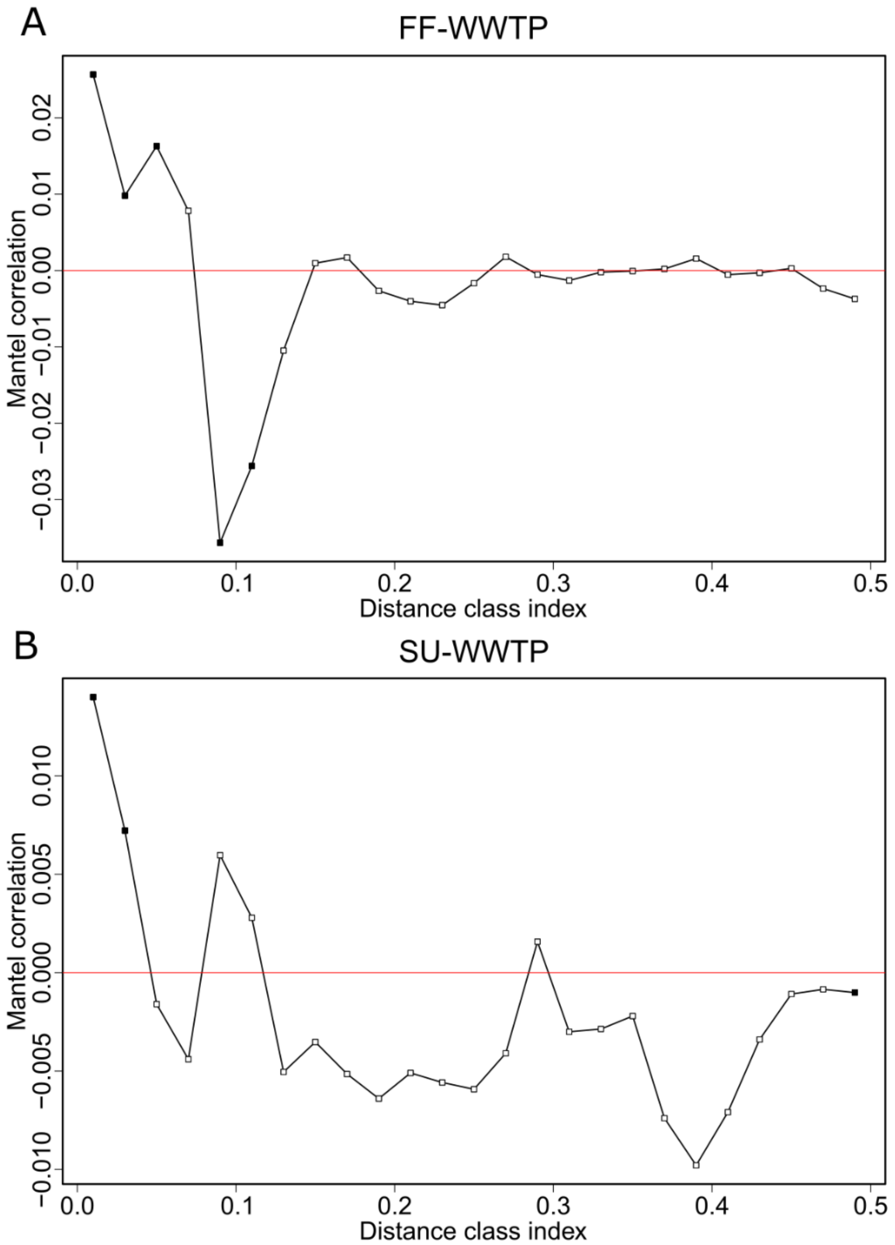

Supplementary Figure 5. Mantel correlograms showing phylogenetic signal. Pearson correlation resulting from Mantel correlogram (999 permutations) between the ASV niche and phylogenetic distances for A) FF-WWTP and B) SU-WWTP. Significant correlations (p value < 0.05, solid quares) indicate phylogenetic signal in species ecological niches.

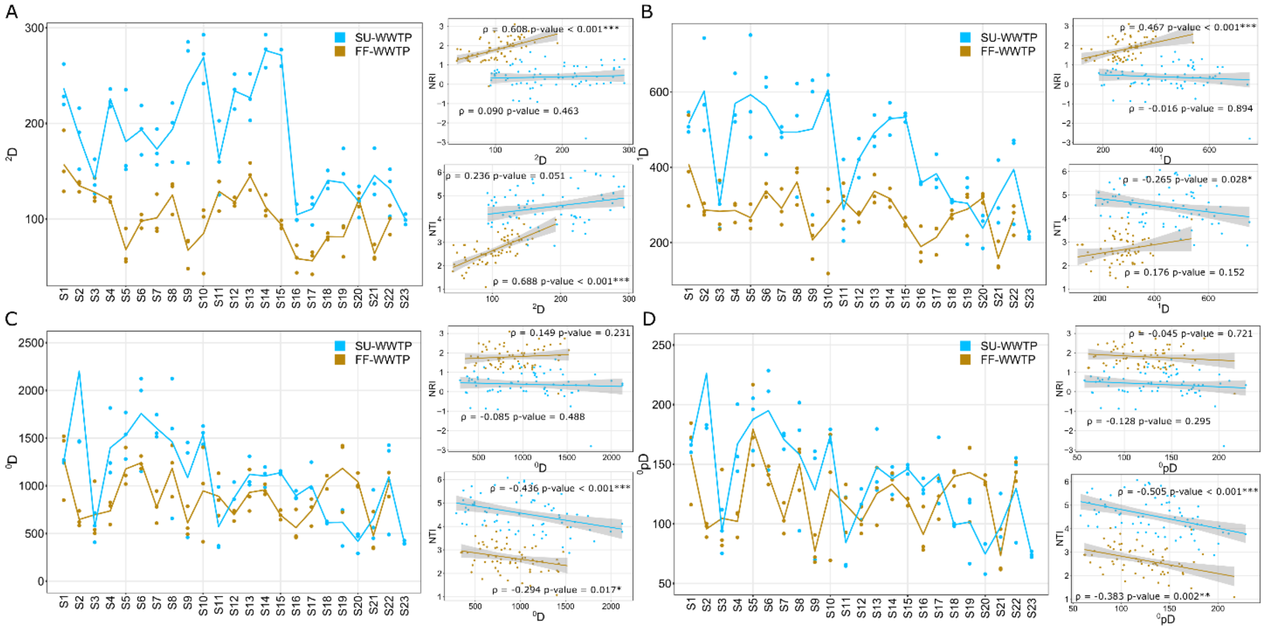

Supplementary Figure 6. Indices of α-diversity and community phylogenetic dispersion against the null expectation. Hill based taxonomic α-diversity accounting for **A**) inverse of dominance, **B**) abundance and **C**) richness, and **D**) phylogenetic α-diversity of diversity order = 0. The left panels correspond with the Spearman's rank correlation between NRI and NTI phylogenetic dispersion metrics and α-diversity metrics. Dots represent sample data (n = 3), and lines represent the average value per time point. Blue points and lines represent SU-WWTP, and brown represent FF-WWTP.
