# Supplementary material for: Niche differentiation drives microbial community assembly and succession in full-scale activated sludge bioreactors
Source: NPJ Biofilms Microbiomes. 2022 Apr 11;8:23. doi: 10.1038/s41522-022-00291-2 (PMC9001656; doi:10.1038/s41522-022-00291-2)
Supplement: Supplementary file 4 — Reporting Summary Checklist [file 41522_2022_291_MOESM4_ESM.pdf]

nature portfolio

Corresponding author(s): IB

Last updated by author(s): Mar 2, 2022

Reporting Summary

Nature Portfolio wishes to improve the reproducibility of the work that we publish. This form provides structure for consistency and transparency in reporting. For further information on Nature Portfolio policies, see our Editorial Policies and the Editorial Policy Checklist.

Statistics

For all statistical analyses, confirm that the following items are present in the figure legend, table legend, main text, or Methods section.

n/a	Confirmed	
☐	☒	The exact sample size (*n*) for each experimental group/condition, given as a discrete number and unit of measurement
☐	☒	A statement on whether measurements were taken from distinct samples or whether the same sample was measured repeatedly
☐	☒	The statistical test(s) used AND whether they are one- or two-sided<br>*Only common tests should be described solely by name; describe more complex techniques in the Methods section.*
☐	☒	A description of all covariates tested
☐	☒	A description of any assumptions or corrections, such as tests of normality and adjustment for multiple comparisons
☐	☒	A full description of the statistical parameters including central tendency (e.g. means) or other basic estimates (e.g. regression coefficient) AND variation (e.g. standard deviation) or associated estimates of uncertainty (e.g. confidence intervals)
☐	☒	For null hypothesis testing, the test statistic (e.g. *F*, *t*, *r*) with confidence intervals, effect sizes, degrees of freedom and *P* value noted<br>*Give P values as exact values whenever suitable.*
☒	☐	For Bayesian analysis, information on the choice of priors and Markov chain Monte Carlo settings
☒	☐	For hierarchical and complex designs, identification of the appropriate level for tests and full reporting of outcomes
☒	☐	Estimates of effect sizes (e.g. Cohen's *d*, Pearson's *r*), indicating how they were calculated

Our web collection on statistics for biologists contains articles on many of the points above.

Software and code

Policy information about availability of computer code

Data collection	n/a
Data analysis	Data analysis has been performed using R version 4.1.0 (relevant packages and algorithms: Dada2, Phyloseq, ggplot2, Vegan, igraph, picante); SINTAX algorithm; MiDAS and Silva databases have been used for taxonomic assignment of ASVs

For manuscripts utilizing custom algorithms or software that are central to the research but not yet described in published literature, software must be made available to editors and reviewers. We strongly encourage code deposition in a community repository (e.g. GitHub). See the Nature Portfolio guidelines for submitting code & software for further information.

Data

Policy information about availability of data

All manuscripts must include a data availability statement. This statement should provide the following information, where applicable:

- Accession codes, unique identifiers, or web links for publicly available datasets
- A description of any restrictions on data availability
- For clinical datasets or third party data, please ensure that the statement adheres to our policy

Raw files are available in the National Center for Biotechnology (NCBI) repository under the project codes PRJNA588045 for FF-WWTP sequences and PRJNA719992 for SU-WWTP sequences.

Field-specific reporting

Please select the one below that is the best fit for your research. If you are not sure, read the appropriate sections before making your selection.

☐ Life sciences ☐ Behavioural & social sciences ☒ Ecological, evolutionary & environmental sciences

For a reference copy of the document with all sections, see nature.com/documents/nr-reporting-summary-flat.pdf

Ecological, evolutionary & environmental sciences study design

All studies must disclose on these points even when the disclosure is negative.

Study description	A total of 135 samples of activated sludge from two different Wastewater treatment plants (WWTP) have been analyzed for bacterial populations description (16S amplicon sequencing).
Research sample	In this study we studied bacterial populations in activated sludge sampled in two different WWTP at different stages of functioning (a starting-up (SU-WWTP) and an already established (FF-WWTP) plant)
Sampling strategy	The 135 swamples analyzed corresponds to triplicate sampling of two time series of 22 and 23 consecutive months in the two studied WWTPs, respectively
Data collection	DNA was extracted using DNA Power Soil extraction kits, libraries were prepared and sequenced on Illumina MiSeq instrument (Illumina®, San Diego, CA, USA) using 2x300 paired-end reads. The 16S-V4 rRNA gene was amplified with the primer set: 515F -GTGYCAGCMGCCGCGGTAA- and 806R -GGACTACNVGGGTWTCTAAT-.<br>Physical-chemical parameters were measured in the influent and the effluent of the WWTP, according to standard methods: biochemical oxygen demand, BOD (UNE-EN-1899); total nitrogen, TN (ISO-11905) and total phosphorous, TP (ISO-6878)
Timing and spatial scale	This study includes samples collected in two different WWTP located in Spain. FF-WWTP samples were taken from April '17 to March '19 (August were not sampled, for a total of 22 samples, n = 3), and SU-WWTP samples were taken from March '17 to February '19 (June '18 was not sampled, for a total of 23 samples, n = 3).
Data exclusions	No available data have been excluded from the analysis
Reproducibility	n/a
Randomization	All the groups established in this study are defined based on the origin (WWTP) and time (month and year) of sampling
Blinding	Three samples per sampling time and WWTP were collected representing different sections of the biological reactors

Did the study involve field work? ☐ Yes ☒ No

Reporting for specific materials, systems and methods

We require information from authors about some types of materials, experimental systems and methods used in many studies. Here, indicate whether each material, system or method listed is relevant to your study. If you are not sure if a list item applies to your research, read the appropriate section before selecting a response.

Materials & experimental systems

n/a	Involved in the study
☒	☐ Antibodies
☒	☐ Eukaryotic cell lines
☒	☐ Palaeontology and archaeology
☒	☐ Animals and other organisms
☒	☐ Human research participants
☒	☐ Clinical data
☒	☐ Dual use research of concern

Methods

n/a	Involved in the study
☒	☐ ChIP-seq
☒	☐ Flow cytometry
☒	☐ MRI-based neuroimaging
